# A multichannel optical computing architecture for advanced machine vision

**DOI:** 10.1038/s41377-022-00945-y

**Published:** 2022-08-18

**Authors:** Zhihao Xu, Xiaoyun Yuan, Tiankuang Zhou, Lu Fang

**Affiliations:** 1 Sigma Laboratory, Department of Electronic Engineering, Tsinghua University, Beijing, China; 2 Beijing National Research Center for Information Science and Technology (BNRist), Beijing, China; 3 Tsinghua Shenzhen International Graduate School, Shenzhen, China; 4 Institute for Brain and Cognitive Science, Tsinghua University (THUIBCS), Beijing, China

**Keywords:** Applied optics, Optical techniques, Transformation optics

## Abstract

Endowed with the superior computing speed and energy efficiency, optical neural networks (ONNs) have attracted ever-growing attention in recent years. Existing optical computing architectures are mainly single-channel due to the lack of advanced optical connection and interaction operators, solving simple tasks such as hand-written digit classification, saliency detection, etc. The limited computing capacity and scalability of single-channel ONNs restrict the optical implementation of advanced machine vision. Herein, we develop Monet: a multichannel optical neural network architecture for a universal multiple-input multiple-channel optical computing based on a novel projection-interference-prediction framework where the inter- and intra- channel connections are mapped to optical interference and diffraction. In our Monet, optical interference patterns are generated by projecting and interfering the multichannel inputs in a shared domain. These patterns encoding the correspondences together with feature embeddings are iteratively produced through the projection-interference process to predict the final output optically. For the first time, Monet validates that multichannel processing properties can be optically implemented with high-efficiency, enabling real-world intelligent multichannel-processing tasks solved via optical computing, including 3D/motion detections. Extensive experiments on different scenarios demonstrate the effectiveness of Monet in handling advanced machine vision tasks with comparative accuracy as the electronic counterparts yet achieving a ten-fold improvement in computing efficiency. For intelligent computing, the trends of dealing with real-world advanced tasks are irreversible. Breaking the capacity and scalability limitations of single-channel ONN and further exploring the multichannel processing potential of wave optics, we anticipate that the proposed technique will accelerate the development of more powerful optical AI as critical support for modern advanced machine vision.

## Abstract (web summary)

We develop Monet: a multichannel optical neural network architecture for a universal multiple-input multiple-channel optical computing based on a novel projection-interference-prediction framework, solving real-world advanced machine vision tasks optically.

## Graphical abstract

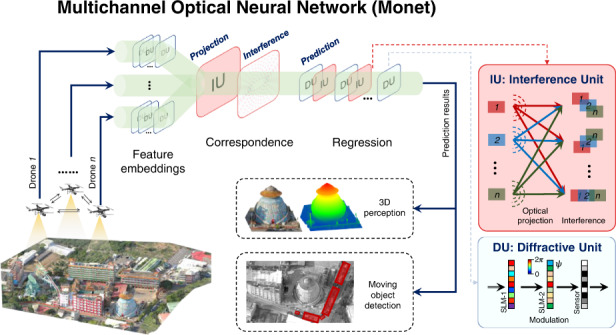

## Introduction

The artificial neural network (ANN) technique has greatly promoted the broad impact of visual computing solutions^1,2^ for machine vision, intelligent robots, autonomous driving, smart city etc. With the extraordinary performance in terms of accuracy and robustness, ANN-based approaches have successfully resolved all sorts of tasks, from fundamental visual processing tasks, e.g., hand-written digit classification^3^ and saliency detection^4,5^, to complex machine vision tasks, e.g., multiview stereo^6–9^ and video processing^10,11^. While developing more and more powerful ANN-based approaches seems never ending, a critical question rises, can existing computing resources support the insatiable computing demand from the ANN? Despite the rapid development of neural processing units (NPUs) in recent years^12–15^, the performance and energy efficiency of conventional silicon-based computing devices are restricted by the plateauing of Moore’s law^16^, leading to the limited scaling of electronic transistors in silicon computing hardware platforms.

As an emerging technology for high-performance computing, all-optical and optoelectronic neural networks (ONNs) have attracted increasing attention in recent years, due to their inherent high speed and high energy efficiency characteristics^17–26^. Fundamental simple visual processing tasks such as hand-written digit recognition^17–24,27^ and saliency detection^20,21^, have been effectively validated using wave-optics simulations or small-scale optical computing systems. Meanwhile, some works combine the optical computing units with a variety of electronic neural networks to enlarge the scale and flexibility of ONNs, e.g., deep optics^28,29^, amplitude-only Fourier NNs^22^, and hybrid optical-electronic CNN^23^. Essentially, these architectures regard optical processing as part of electronic networks, resulting in a failure to fully take advantages of optical computing. Recently, researchers start to multiplex optical computing units^20,30^, achieving a much higher proportion of optical computing and better computing performance, e.g., diffractive processing unit (DPU)^20^ reaches superior computing performance compared to the state-of-the-art electronic computing platforms in specific neural network inference.

Despite the aforementioned ONN progresses, many widely existing complex machine vision tasks such as 3D detection or video processing, which are thirsty for network scaling and computing resources, have not been solved with ONNs^18,27,31^. Due to the lack of advanced optical connection and interaction operators, existing ONNs mainly remain single-channel feedforward architectures, solving primary single-image processing tasks. However, as widely verified by ANNs from task-specific multiple-input neural networks^32–35^ to general convolutional neural networks^36–39^, multichannel and multi-input processing ability lays the foundation of advanced machine vision. Simply enlarging the scale (number of neurons) within the single-channel-based architectures can hardly increase the computing capacity to meet the demand of advanced machine vision tasks, as “quantitative change does not necessarily cause qualitative change”. Directly combining optical computing units with a large proportion of electronic computing units may deal with complex tasks^20,30^, however, such straightforward and compromising solution cannot fully exploit the physical nature of the light, either failing to maintain high efficiency of the optical computing, or sacrificing the abundant optical information carried by the intrinsic properties of light, e.g. phase. Undoubtedly, developing fundamental multichannel processing methods for realizing more powerful ONNs remains elusive.

**Fig. 1:**
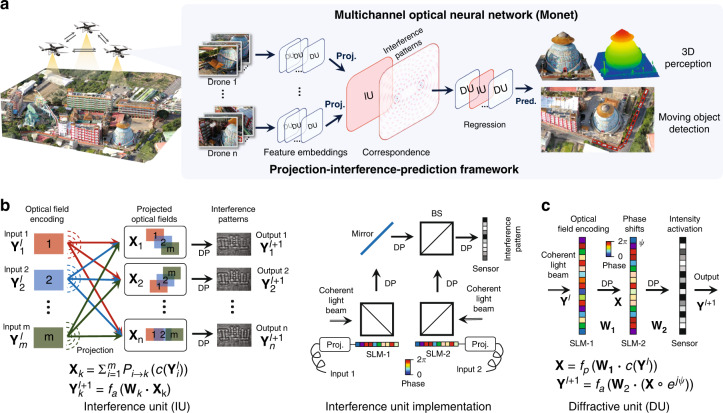
A multichannel optical neural network (Monet) architecture for advanced machine vision tasks. **a** Network architecture of Monet and the projection-interference-prediction framework. Multiple observations are projected to a shared domain and encoded into optical fields, processed by interference units (IUs) for correspondence constructions and diffractive units (DUs) for feature embeddings. A regression module composed of iteratively connected IUs and DUs is adopted to predict the results for 3D perception or moving object detection. **b** Schematic and physical implementation of the IU (two-input). Multiple optical fields encoding multiple images are projected by task-specific function, propagate, and interfere to generate interference patterns. Different colours (red, green, blue) denote different visual inputs. In the physical implementation, two spatial light modulators are used to generate and project the optical fields, and a sensor is used to capture the interference pattern. **c** Schematic of the DU. A coherent light beam is modulated by SLM-1 to encode the input image, propagates to the SLM-2 for modulation, and propagates to the sensor for activation. DP diffractive propagation, BS beam splitter, Proj projection, Pred prediction

Herein, we report a multichannel optical computing architecture for advanced machine vision (Fig. 1), in which optical interference and diffraction are utilized to establish inter- and intra- channel connections, respectively. Considering the physical propagation of coherent optical fields, constructive and destructive patterns arise from spatial interference. These patterns, in turn, carry rich information of inter-channel connections. Meanwhile, wavefront modulations are adopted during the diffraction of optical field propagation for the intra-channel connections. On the basis of these connections, we innovatively develop a projection-interference-prediction framework with iteratively deployed optical interference units (IUs) and optical diffractive units (DUs) to establish *Monet: a multichannel optical neural network*. In this framework, multichannel inputs are projected to a shared spatial domain, aligned with a series of predefined overlapping strategies and interfered to form patterns containing rich correspondence encodings in IUs. Subsequent DUs perform optical diffraction computing for feature embeddings from these interference patterns. As novel optical connection and interaction operators, the collaborated IUs and DUs produce patterns encoding the correspondences together with feature embeddings layer by layer through the projection-interference process to predict the final output. It is worth noting that unlike existing ONNs trying to inherit ANN architectures, the projection-interference-prediction framework of Monet is initially designed following the innate characters of light propagation and interaction, to further explore the multichannel processing potential of wave optics in optical computing.

**Fig. 2:**
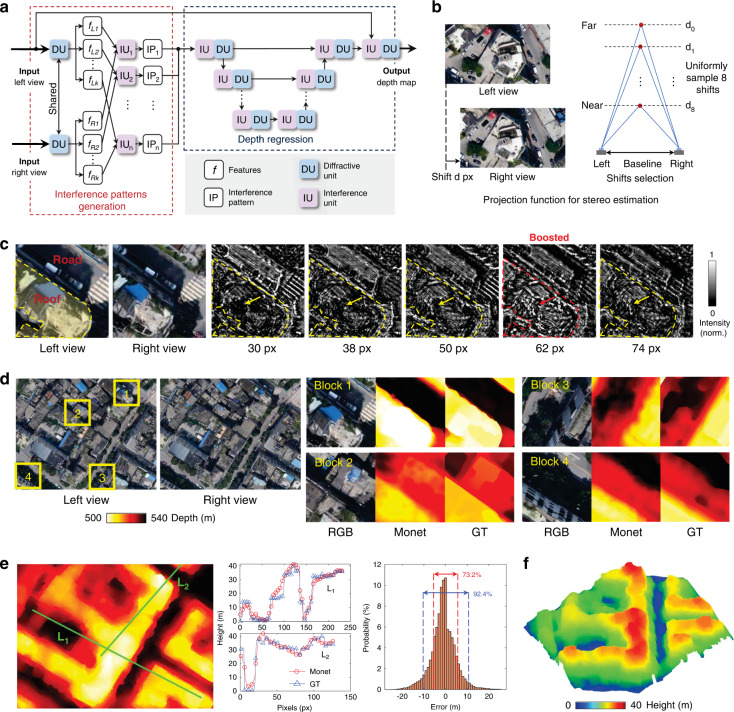
Stereo depth estimation results. **a** Network architecture of the Monet for stereo depth estimation, composed of an interference pattern generation module followed by a depth regression module. The former module encodes the stereo correspondence into the interference patterns, and the latter module adopts a U-net-like structure to estimate the depth information from the patterns. **b** Projection function for stereo depth estimation. The spatial position of the left-view image is fixed and the right-view image is shifted to the right according to the depth range. **c** Interference patterns with 5 shift distances (presented below each interference pattern). **d** A large area from the test set. Stereo depth estimation results of 4 representative blocks (labelled using yellow boxes) are shown on the right. **e** Stitched depth map of the large area in subfig. **d** The depth value is converted to the height relative to the ground for a more intuitive understanding. The heights along two lines L_1_ and L_2_ are presented in the middle. The depth-error distribution of the whole area is shown on the right. **f** Height-coloured 3d model of the large area. Norm normalized, GT ground-truth, px pixel

**Fig. 3:**
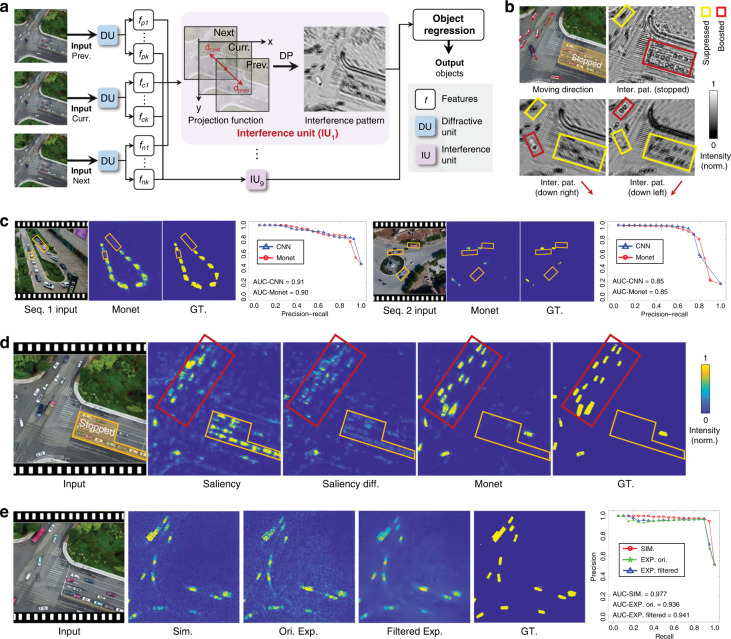
Moving object detection results. **a** Network architecture and the projection function of the Monet for moving object detection. The previous frame, current frame and next frame are used as three inputs of Monet. The previous frame and next frame are shifted d_prev_ and d_next_ pixels, and the current frame is fixed in the projection function. **b** Interference patterns of a representative frame. The objects are boosted when the projection direction is close to the ground-truth moving direction (labelled with red boxes), while the objects with orthogonal moving directions are suppressed (labelled with yellow boxes). **c** The input sequences, Monet computational outputs and ground-truth of two representative sequences. The stopped objects are labelled using orange boxes, which are successfully suppressed. Object-level precision-recall (PR) curves and the areas under the curves (AUCs) of each sequence are also presented. An electronic CNN is used for comparison. Note that the moving objects are highlighted in the outputs of Monet, showing very high similarity with the GT. **d** Comparison results between Monet and the existing optoelectronic saliency detection approach^51^. Saliency diff. shows the difference of the next-frame and the previous-frame saliency maps. **e** The input sequence, Monet simulation results, original and bilateral filtered physical experimental results, and ground-truth of a representative frame in the test sequences. The object-level PR curve and AUC are illustrated on the right. The outputs of Monet simulation and optical Monet experiments show a high correlation in the final distribution. The optical output shows acceptable performance loss compared with simulations. With a simple bilateral solver applied in the final results, the speckles are removed for better visualization. Prev previous, Curr current, Seq sequence, Inter interference, Pat pattern, Norm normalized, GT ground-truth, Sim simulation, Ori original, Exp experimental, Diff difference, DP diffractive propagation

**Fig. 4:**
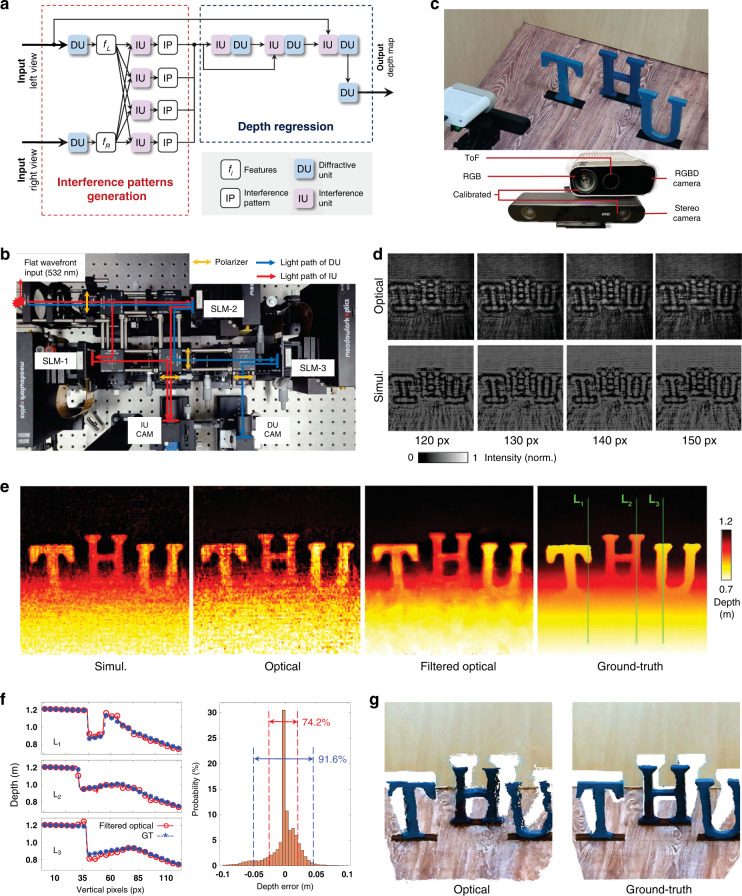
Prototype system of Monet for real-world experiment. **a** Monet network architecture used in the prototype system implementation. **b** Prototype system built with off-the-shelf optical modulation devices. The red-light path denotes the IU and the blue-light path denotes the DU. **c** Top, a customized scene with three character-shaped objects ‘T, H, U’. Bottom, the hybrid camera system for dataset capturing. **d** Optical and simulated interference patterns with 4 different shifts. The number below each column denotes the shift distance of the interference pattern. **e** Depth maps of numerical simulation, optical result (raw output of the prototype system), filtered optical result and the ground-truth. **f** Left, depth values along three lines L_1_, L_2_ and L_3_ on the filtered optical result and the ground-truth. Right, the depth-value error distribution of the whole image. **g** Point clouds generated from the filtered optical results and the ground-truth. SLM spatial light modulator, CAM camera, IU interference unit, DU diffractive unit, Simul. simulation, Px pixel, GT ground-truth

In the experiments, for the first time we validate that multichannel processing properties can be effectively implemented in intelligent optical computing, enabling advanced machine vision tasks such as 3D detection (Fig. 2) and moving objection detection (Fig. 3) can be accomplished using Monet with excellent performance. Moreover, we develop the optical prototype system of Monet using off-the-shelf optical modulation devices. The physical implementation of Monet’s multichannel architecture demonstrated its motion detection ability (Fig. 3e) and 3D detection ability (Fig. 4) in real-world scenarios. Oriented from the wave properties of light itself, our Monet gets rid of the common practices in conventional electronic or optical neural network architectures, leading to a more natural and practical way for optical computing to achieve multichannel processing abilities and thus overcome the insufficient-capacity problem of previous ONNs in complex machine visions.

Collectively, the experiments of Monet imply the great potential of ONN in processing complex visual inputs and tasks, e.g., given several video sequences captured by multiple drones, ONNs may intellectually infer depth information and detect moving objects with advantageous speed and energy efficiency compared with commercial GPUs. (See Supplementary Note 3 for detailed energy efficiency analysis). We expect that our study will pave a new way for optical computing from the early stage of simulations to a new era of solving real-world complex tasks practically, leveraging the rapid growth of computing resources demand.

## Results

### The multichannel optical neural network (Monet)

Figure 1a illustrates the network architecture of Monet, including interference units (IUs, Fig. 1b) for inter-channel processing and diffractive units (DUs, Fig. 1c) for intra-channel processing. Regarded as novel optical connection and interaction operators, the IUs and DUs further collaborate in the projection-interference-prediction framework: multiple observations are encoded to coherent optical fields, projected to a shared domain for feature embeddings through DUs, and for correspondence constructions through IUs. A regression module composed of iteratively connected IUs and DUs is followed to predict the results from the interference patterns for 3D perception or moving object detection. Figure 1b illustrates the schematic and physical implementation (dual-input) of the interference unit. ${{{\mathbf{Y}}}}_1^l$,${{{\mathbf{Y}}}}_2^l$,…,${{{\mathbf{Y}}}}_m^l$ are the m inputs of layer *l*, and ${{{\mathbf{Y}}}}_1^{l + 1}$,${{{\mathbf{Y}}}}_2^{l + 1}$,…,${{{\mathbf{Y}}}}_n^{l + 1}$ are the n output interference patterns. The inputs are encoded into optical fields, projected to a shared domain, and propagate to generate interference patterns. In the physical implementation, SLMs are used to encode and project the optical fields, and a sensor is used to capture the interference patterns. Projection can be achieved by adding phase shifts on the encoded optical fields or directly changing the modulation patterns of the SLMs. *P*_*i*→*k*_(·) denotes the projection function from the i-*th* input channel to the k-*th* interference pattern, *c*(·) denote the encoding function of the SLM, and **W**_*k*_ is the diffractive propagation matrix from the SLM plane to the sensor pattern.∘ denotes the element-wise product, and · denotes the matrix multiplication. *f*_*a*_(·) is the activation function of the sensor (conversion from complex optical field to intensity). The encoding method *c*(·) can map the inputs to the amplitude or phase domain of the optical field by tuning the polarization of the light beam (see Supplementary Fig. S11), aiming for image fusion or correspondence construction, respectively.

More specifically, in image-fusion IU, amplitude encoding is used, and the optical intensity captured by the sensor reflects the weighted sum results of the input images. The weights can be adjusted using polarizers or optical attenuators. The image-fusion IU is mainly used in the regression module to achieve inter-channel processing (see Supplementary Figs. S1, S2 and S3, fusion module). It increases the width (number of neurons) in a single layer, which is as important as the depth of the neural network^40,41^. In correspondence-construction IU, phase encoding is used and the interference patterns are captured by the sensor to accumulate the differences among all the inputs (see Supplementary Note 1 for detailed derivation):

$$\begin{array}{lll}{{{\mathbf{Y}}}}_{inter} =\, | {e^{j{{{\tilde{\mathbf Y}}}}_1} + e^{j{{{\tilde{\mathbf Y}}}}_2} + \ldots + e^{j{{{\tilde{\mathbf Y}}}}_N}}|^2 \\\qquad\quad\,=\, 2\mathop {\sum}\nolimits_i^N {\mathop {\sum}\nolimits_{j + 1}^N {\cos \left( {{{{\tilde{\mathbf Y}}}}_i - {{{\tilde{\mathbf Y}}}}_j} \right) + N} }\end{array}$$

$${{{\mathbf{Y}}}}_{inter}(N = 2) = | {e^{j{{{\tilde{\mathbf Y}}}}_1} + e^{j{{{\tilde{\mathbf Y}}}}_2}}|^2 = 2\cos \left( {{{{\tilde{\mathbf Y}}}}_1 - {{{\tilde{\mathbf Y}}}}_2} \right) + 2$$

where ${{{\tilde{\mathbf Y}}}}_1$, ${{{\tilde{\mathbf Y}}}}_2$,…,${{{\tilde{\mathbf Y}}}}_N$ denote the projected optical fields encoding the multiple inputs, and **Y**_*inter*_ denotes the captured interference pattern. Thus, by appropriately designing the projection function, the correspondences among multiple inputs can be converted to per-pixel differences, encoded to interference patterns, and utilized by the followed regression module. Correspondence construction aims to find the similarity among multiple inputs, serving as a critical step for advanced multichannel machine vision tasks. For the stereo depth estimation task (Fig. 2), we fix the spatial position of the left-view image and shift the right image horizontally multiple times to generate the interference patterns which encode the stereo matching cost. For the moving object detection task (Fig. 3), we fix the spatial position of the current-frame image, and shift the previous- and next-frame images along with multiple directions to generate interference patterns for finding objects with certain moving directions.

Figure 1c shows the implementation of DU. **Y**^*l*^ is the input of layer *l*, *c*(·) maps the input to optical fields using a spatial light modulator (SLM-1), **W**_1_ represents the diffractive propagation matrix from SLM-1 to SLM-2, **X** denotes the optical field before SLM-2, and *f*_*p*_(·) denotes the polarizer to align the optical polarization of **X** with the fast axis of SLM-2. **X** is modulated and propagates to the sensor as the output of the DU. *ψ* denotes the trainable phase shifts introduced by SLM-2, **W**_2_ represents the diffractive propagation matrix from SLM-2 to the sensor, and *f*_*a*_(·) denotes the activation function of the sensor. The DU can be treated as a neural network layer and the modulation phase *ψ* can be optimized by back-propagation.

### Stereo depth estimation

Stereo depth estimation is the most fundamental and important task of 3D perception^42,43^. We design a dual-input Monet architecture for stereo depth estimation (Fig. 2a) and verify it on the WHU Stereo dataset^44^ (see Methods for dataset preparation). The network can be divided into two modules: interference pattern generation and depth regression. In the former module, two sets of DUs with shared weights are first used to extract features from the left-view and right-view images, and IUs are followed to generate the interference patterns. The projection function is presented in Fig. 2b: We fix the position of the left-view image and shift the right-view image *d* pixels to the right. Here, we uniformly sample 8 shift distances in the range from the farthest disparity *d*_0_ to the nearest disparity *d*_8_, and generate 8 interference patterns. These patterns are then input to the depth regression module, which adopts a U-net-like structure^45^ composed of iteratively connected IUs and DUs. See Supplementary Fig. S1 and S2 for more details.

Figure 2c illustrates 5 representative interference patterns. Image regions whose ground-true disparity is close to the shift distance are boosted in the interference patterns, while the remaining regions are suppressed. The roof (disparity = 60 px) in Fig. 2c is boosted in the interference pattern with a 62-px shift (labelled red) and suppressed in that with 30-px and other shift values (labelled yellow). Figure 2d illustrates a large area (1406 × 1052) in the test set, which is divided into 30 stereo blocks with 350 × 350 for Monet processing (see Methods). The depth estimation results of 4 representative blocks are shown on the right, and their positions are labelled using yellow boxes on the left-view image. Monet successfully predicts depth variation among the buildings, trees, and roads, and the estimated depth maps show very similar structures to the ground-truth. Figure 2e shows the stitched depth map of the whole large area. Bilateral solver^46^ is used to suppress the block effects between the adjacent blocks. The ground is approximately 550 m from the drone. We convert the depth value to the height relative to the ground for a more intuitive understanding, and plot the height along two lines L_1_ and L_2_ in the middle. These two curves correlate well, with errors of 2.6 ± 2.4 m (L_1_, mean±s.d.) and 2.8 ± 2.7 m (L_2_, mean ± s.d.), respectively. The depth-value error of the whole area is 4.2 ± 3.9 m (mean ± s.d.), and the error distribution is shown on the right: 73.2% of pixels lie in (−6 m, 6 m), and 92.4% of pixels lie in (−11 m, 11 m). The quantitative evaluation results prove the depth estimation ability of Monet. We further design an extra-shift experiment to show that Monet predicts the depth information from the disparity of the stereo images, not just learning a style transferring or monocular depth estimation model. In particular, we fix the spatial position of the left-view images (test set only), and apply a series of extrashifts on the corresponding right-view images to form a new test set. The output on the new test set shows that Monet can predict the correct disparity values with extrashifts (see Supplementary Fig. S5). The height-coloured 3D model of this area is presented in Fig. 2f. The structures of the buildings are well reconstructed. See Supplementary Fig. S4 and Supplementary Video 1 for more results.

### Moving object detection

Moving object detection tasks are widely existed in various machine vision applications, e.g., smart city and road condition monitoring^47–49^. We implement moving object detection using Monet to show that our architecture can process image series and extract temporal semantic information. Figure 3a illustrates the network architecture and the projection function. Similar to stereo depth estimation, the whole network also consists of an interference pattern generation module and an object regression module.

To detect the moving objects in the current frame, three channels (previous, current, and next frames of a video) are used as the inputs. We remove the skip connection from the input image to the regression module as this task does not require sharp-edge outputs. The projection function is extended to 2 directions to detect the moving objects in both directions. As shown in Fig. 3a, we fix the spatial position of the current frame, and shift the previous frame and next frame for *d*_*prev*_ and *d*_*next*_ pixels, respectively. Three encoded optical fields then propagate and interfere on the sensor plane to generate patterns. To compensate for the movement of the camera/drone, images are registered before input to Monet (see Methods). The object regression module also adopts a U-net-like structure to predict the moving object distribution maps from the interference patterns. See Supplementary Figs. S1 and S3 for network architecture details.

We validate our Monet on the VisDrone dataset^50^ (see Methods for dataset preparation). Figure 3b demonstrates 3 representative interference patterns with stopped (*d*_*prev*_ = *d*_*next*_ = (0,0)), down-left (*d*_*prev*_ = (7,7) px, *d*_*next*_ = (−7,−7) px) and down-right (*d*_*prev*_ = (−7,7) px, *d*_*next*_ = (7,−7) px) projection directions. The top-left image in Fig. 3b shows the moving directions of the objects. Objects with moving directions close to the projection directions are boosted in the interference pattern (labelled using red boxes), while the objects with orthogonal moving directions are suppressed (labelled using yellow boxes). Figure 3c demonstrates 2 representative sequences in the test set with both moving and stopped objects. The stopped objects (labelled using orange boxes) are suppressed in the outputs of Monet, while the moving objects are successfully preserved (see Supplementary Video 2 for more results). We compare our Monet with an electronic convolution neural network with a similar network architecture^51,52^ (denoted as CNN, see Supplementary Fig. S3). The object-level precision-recall (PR) curves of these 2 sequences are presented in Fig. 3c (see Methods for curve computation). Both Monet and CNN achieve high AUCs.

To validate the moving object detection ability of Monet in real optical system, we build a prototype physical system using off-the-shelf optical components, including spatial light modulators (SLM), beam splitters, laser source, and sensors (see Methods for system constructions). To reduce the influences caused by the imperfectness of optical components in the real optical system, we reduce the scale of dataset in network training and simplify the network scale from 8 layers to 5 layers (see Supplementary Fig. S3 for detailed network structure). Although the output regression part is simplified for the ease of experiment, the core innovation, the shifting interference part remained unchanged. Figure 3e shows the Monet simulation results, original and filtered Monet physical experimental results of a representative sequence. Due to the laser properties and the imperfectness of optical components, we can find noise in the original physical outputs. The noise does not affect the performance much as the object level PR curve and the AUC metrics show close accuracies. We further apply a bilateral solver for better visualization (bilateral solver only takes very little computing resource, see Supplementary Fig. S8 for detailed results in physical experiments and Supplementary Fig. S9 for the comparison of pixel-level PR curves and pixel-level AUCs). To suppress the noise and match the spatial resolution of the intermediate optical computing results, we apply pixel binning operation provided by the sensor and then warp the down-sampled sensor images to the SLM patterns using precalibrated homography matrix (see Supplementary Fig. S12 for detail in sensor-SLM calibration). In this way, we could achieve similar results in both computational simulation results and optical system outputs (see “Optical regularization methods” in the Methods section for more details).

Figure 3d compares our Monet with f-D^2^NN, an previously published ONN for saliency detection^21^. Restricted by its single-channel processing framework, it fails to distinguish stopped objects from moving objects. The difference between the next-frame and previous-frame saliency map (Saliency diff.) shows that simple subtraction cannot filter out the moving objects well. Although the stopped objects (orange box) are suppressed, the objects with relatively small movements are also mistakenly suppressed (red box). Moreover, the shapes and numbers of the moving objects become almost unrecognizable in the difference map (red box). While our Monet can effectively detect moving objects and maintain their shapes very well.

### Prototype system of Monet for real-world experiment

We develop the Monet prototype system using off-the-shelf optical modulation devices (Fig. 4b) and test its 3D perception capability on a customized indoor scene with 3 character-shaped objects ‘T, H, U’ (Fig. 4c). The network architecture implemented by our prototype system is presented in Figs. 4a (see Supplementary Fig. S1 and S2 for detailed network architecture). An online training framework is applied to overcome the non-ideal characteristics of the laser and optical modulation devices (see Methods and Supplementary Fig. S10). Figure 4b illustrates our prototype system. A 532-nm continuous-wave laser is used to generate the flat wavefront. The IU (red light path) consists of two SLMs and the IU camera. Two SLMs (SLM-1 and SLM-2) are used to encode the dual inputs into optical fields. The DU (blue light path) is composed of two SLMs and the DU camera. SLM-2 is used to encode the input, and SLM-3 is used to apply the phase modulation. See Supplementary Figs. S12, S13 and S14 for the configuration of the polarizers and the calibration of the SLMs. A hybrid camera system composed of a stereo camera and an RGBD camera is designed to capture the stereo images and the ground-truth depth maps of the dataset (Fig. 4c bottom; see Methods and Supplementary Fig. 15 for more details).

The optical (prototype system) and simulated interference patterns are presented in Fig. 4d. Due to the unavoidable physical error in the prototype system, the small interference fringes of the optical results are different from the simulated ones, but the edges of the three character-shaped objects and the overall changes with increasing shifts are very similar. For example, the edges of ‘U’ are blurry in the interference pattern of the 120-px shift but become focused and clear in the interference pattern of the 140-px shift. Figure 4e illustrates the simulation results, optical results (prototype system), filtered optical results (bilateral solver^46^ is used to remove the speckles) and ground-truth depth maps. Our prototype system successfully estimates the depth differences within and among the character-shaped objects. ‘T’ and ‘U’ are placed at the same depth while ‘H’ is placed behind them. The upper part of the character-shaped object is closer to the camera than the bottom part. The depth-value changes along three lines L_1_, L_2_, and L_3_ are plotted in Fig. 4f. Three curves of the filtered optical depth maps correlate well with the ground-truth, with errors of 0.017 ± 0.025 m (L_1_, mean ± s.d.), 0.011 ± 0.013 m (L_2_, mean ± s.d.) and 0.018 ± 0.021 m (L_2_, mean ± s.d.), respectively. The error distribution of the whole image is illustrated on the right. 74.2% of pixels have errors less than 0.045 m, and 91.6% of pixels have errors less than 0.095 m. We further back-project the depth maps to 3D point clouds for better visualization (Fig. 4g). More results including the intermediate feature maps and the 3D rendering of the point clouds are displayed in Supplementary Fig. S11 and Supplementary Video 3.

## Discussion

This work innovatively develops a multichannel optical neural network (Monet) for advanced machine vision tasks. The proposed projection-interference-prediction framework is built by multiplexing the optical interference units and diffractive units, which can be constructed using off-the-shelf optical modulation devices and sensors. SLMs are used to modulate the phase of optical fields to implement intra- and inter-channel optical computing. Sensors combined with the multiplex strategy act as nonlinear activation functions. Note that all the computations are conducted using optics except nonlinear activation. At present, the speed of our prototype system may be restricted by the multiplexing of the SLM (HDMI-interface, maximum 60 fps) and the sensor (USB 3.1 interface, maximum 75 fps). It is promising to further integrate Monet into optical AI chips by replacing the off-the-shelf devices, e.g. SLMs and sensors, with fabricated phase masks and non-linear optical materials such as SBN:60^53–55^, which would not only overcome the delay caused by the multiplexing of SLMs and sensors but also dramatically reduce the volume of the system.

Correspondence construction among multiple inputs is the foundation of multi-channel processing tasks especially in advanced machine vision, such as multiview stereo, video processing, and volume data processing. Monet implements the inter-channel correspondence for stereo images and video sequences through phase-modulation interference units. Unlike conventional electronic neural networks which commonly adopt the ‘concatenate’ operation to merge information from multiple channels, interference among multiple phase-modulated optical fields has the innate ability to encode the correspondence directly and optically. Thus, by designing task-specific projection strategy and taking advantage of this inherit characters of light propagation and interaction, our Monet architecture paves its own way to fully explore the potential of wave optics for optical computing.

For ease of demonstration, the presented tasks take two- or three-images as input. However, the proposed Monet with projection-interference-prediction framework can be directly extended to support more inputs and more complex visual computing tasks by designing appropriate projection functions. E.g., besides the used projection functions of image shifts, more advanced projection functions, including homography transformation and feature embedding, can be completed using customized optics or configurable optical modulation devices.

For intelligent computing, the trends of dealing with more advanced tasks are irreversible. As a novel technique, Monet implies great potential of ONNs in processing complex visual inputs and tasks, enabling the real-life applications of optical computing, e.g., given a number of video sequences captured by unmanned system, ONNs may directly infer the 3D depth map and detect moving objects with high speed and low power consumption. We anticipate that the proposed technique will accelerate the development of more powerful optical AI as critical support for modern advanced machine vision and towards beginning a new era of AI.

## Methods

### Monet prototype system design

A continuous 532-nm laser (MGL-FN-532, Changchun New Industries Optoelectronics Technology Co., Ltd) was adopted as the light source. A 4-f-system-based 10× optical beam expander (25-mm and 250-mm lenses) was used to generate the flat wavefront. Liquid-crystal-on-silicon (LCOS) SLMs (E-Series 1920 × 1200, Meadowlark Optics Inc., USA) were used to modulate the phase of the wavefronts. The SLMs were calibrated at 532-nm wavelength, with an 8-μm pixel size and 1920 × 1200 resolution, and controlled by the HDMI port. Two grayscale CMOS cameras (Blackfly S BFS-U3-51S5M-C, FLIR LLC, USA) were used to capture the outputs of the IUs and DUs. The camera resolution is 2448 × 2048 with a pixel size of 3.45 μm. The small pixel size ensures that the high-frequency content on the sensor plane can be captured without aliasing. The camera was set to a 27-μs exposure time and 0-dB gain, and the gamma correction was set to 1. For the IU, the propagation distance from the SLM to the sensor plane was set to 200 mm. For the DU, the propagation distance from the first SLM (input encoding) to the second SLM (modulation) was set to 400 mm, and the distance from the second SLM to the sensor was set to 200 mm. A regular pattern was trained to calibrate the relative spatial position of the two SLMs and the sensor (Supplementary Fig. S12). All the SLMs and sensors were controlled using self-developed Python scripts.

### Hybrid camera system and THU dataset capturing

The THU dataset was captured by a hybrid camera system composed of an RGBD camera (Azure Kinect, Microsoft Corp., USA) and a stereo camera (ZED 2, StereoLabs Inc., USA). The RGB camera inside the Azure Kinect was calibrated with the two RGB cameras of ZED 2. The ToF depth camera inside the Azure Kinect was pre-calibrated to its RGB camera before leaving the factory. Thus, the accurate depth maps captured by the ToF camera inside Azure Kinect can be mapped to the ZED 2 cameras and used as the ground-truth. See Supplementary Fig S15 for calibration details. Due to the viewpoint difference between the two cameras and the imperfect reflection of the scene objects, there were holes in the remapped depth maps. An inpainting algorithm^56^ was used to fill the holes. Both the ‘T, H, U’ character-shaped objects and the background box were made of wood. The character-shaped objects were painted blue, and the bottom of the box was covered with woodgrain wallpaper.

### Neural network modelling and training

Our network implementation consists of four main basic layers: free-space propagation, SLM modulation, sensor, and remapping. The IUs and DUs are built with these basic layers. The simulation pixel size is set to 8 *μm* (same as the physical pixel size of the SLM). The free-space diffraction propagation layer is modelled using the angular spectrum method, and the x-y plane size is set to 800 × 800. Zero padding is implemented to guarantee the boundary condition of the free-space propagation. The SLM modulation layer adds phase shifts to the input optical field. The trainable neuron number is also set to 800 × 800 (6.4 × 6.4 mm, 0.64 million parameters). The sigmoid function is used to constrain the phase-modulation range to 0-2π for training. As the polarizer after SLM changes the amplitude of the optical field, the phase-to-intensity mapping curve of SLM is calibrated and fitted using 10^*th*^-order polynomial functions (see Supplementary Fig. S14). Thus, the phase-to-intensity mapping can be modelled as a differentiable function for back-propagation. The sensor layer converts the complex optical field (amplitude and phase) to the intensity field. As we set the gamma correction to 1, the intensity-to-pixel-value mapping is linear. The remapping layer converts the normalized intensity field (divided by the maximum intensity) back to the complex optical field as the inputs for the IUs or DUs. Two kinds of remapping layers, phase remapping and amplitude remapping, modulate the information to the phase and amplitude domains of the output optical field for correspondence construction and image fusion, respectively.

The total parameters and layers of our network architectures are 26.2 million (10 layers), 26.2 million (10 layers), and 4 million (6 layers) for stereo depth estimation of aerial images (Fig. 2), moving object detection (Fig. 3) and the prototype system (Fig. 4). As the input image and the ground-truth sizes are smaller than 800, zero padding is implemented. For stereo depth estimation (Figs. 2 and 4), a mixed mean squared error (MSE) and structural similarity index metric (SSIM) loss is used:

$$L = 0.8 \ast {{{\mathrm{MSE}}}} + 0.2 \ast (1 - {{{\mathrm{SSIM}}}})$$

Both the output and groundtruth are normalized to 0-1, so that the output ranges of MSE and SSIM are also 0-1. The SSIM loss here is to suppress the speckles and noise. For moving object detection, soft-intersection-over-union (soft-IoU) loss is adopted^57^. Compared with the MSE loss that was used by all-optical saliency detection^21^, soft-IoU loss can achieve a faster convergence speed and better performance.

The network model is implemented using TensorFlow V2.4 (Google LLC) running on a desktop computer (Nvidia GTX 1080 Ti 11 G, Intel i7-6800K CPU with 6 cores, 128 GB RAM and the Microsoft Windows 10 operating system). The network parameters were optimized using the Adam optimizer^58^. Due to the physical errors introduced in the prototype system, the neural network parameters need to be fine-tuned online. We experimentally recorded the outputs of the previous layers (DUs and IUs) and took the physically captured results to train the followed-by layers (Supplementary Fig. S10). We also calibrated the energy distribution of the input laser illumination to compensate for the background non-uniformity of the sensor-captured images (Supplementary Fig. S13).

### Dataset preparation

We use the WHU Stereo dataset^44^ for the aerial-image stereo depth estimation task (Fig. 2). The original dataset was re-rendered from a 3D digital surface model produced from thousands of real aerial images, covering an area of 6.7 × 2.2 km^2^, containing buildings, factories, rivers, roads, and trees. As the image sizes of the original dataset are too large to be directly processed, we selected several large images (5736 × 5736) as our new dataset and cropped them into tiles of 350 × 350 to match the input size of Monet. We further deleted the images with few textures or incorrect depth labels, and finally, we had 340 training samples and 120 testing samples. The testing images cover 4 representative large areas with a size of 1406 × 1052, and each area was divided into 30 350 × 350 blocks. The testing stereo images were sampled densely with large overlaps, so that the estimated depth maps could be stitched back to the large area. The training images were cropped from the remaining regions with small overlaps.

The VisDrone detection dataset^50^ is used for moving object detection (Fig. 3). This dataset contains aerial-view videos captured by a drone with no or slow motion. Bounding boxes of all the objects (mainly persons and vehicles) are given in the ground-truth labels. To find the moving objects, we first registered the video frames using SIFT feature points^59^ extracted from the background, and used the centers of the bounding boxes to track the moving distance of all the objects. Feature points on the moving objects were removed before estimating the homography matrices between consecutive frames. Objects with moving distances smaller than the threshold were treated as static objects. After determining all the moving objects, we applied the Grabcut^60^ algorithm to generate the masks from the bounding boxes. We also manually refined the regions in which Grabcut failed to estimate reasonable masks. As the aspect ratio of VisDrone videos is 16:9 while our network prefers square inputs, we split each sequence into a left sequence and a right sequence to make full use of the dataset.

The last dataset used is the THU dataset we captured (Fig. 4). Sixty-five stereo images with different ‘T’, ‘H’, ‘U’ positions were captured in total, 56 for training and 9 for testing. Similarly, the stereo images and depth maps were resized to 700 × 700 to match the network input.

### Precision-recall curve

The PR curve was computed from the moving object distribution maps output by Monet. Two kinds of PR curves were computed: pixel level and object level. In pixel-level PR curve computing, each pixel was considered as an instance. The absolute pixel value was used to determine if the pixel was positive or negative (see Supplementary Fig. S7 for pixel-level PR curves). In object-level PR curve computing, each object was considered as an instance. For each instance, we computed the mean pixel value of the pixels inside the groundtruth object mask (both moving and static objects). If the mean pixel value exceeded the threshold, the object was considered positive; otherwise, it was considered negative.

### Optical regularization methods

In our optical Monet experiment, the visual noise in the results is not only caused by the laser properties, but also resulted by the little mismatch between SLMs and other optical devices. The pixel size of the SLM display is micrometer level and such alignment accuracy is difficult to achieve manually. Once mismatch exists, the noise emerged. Our method to handle it is to regularize the smoothness of the SLM patterns by employing binning and Gaussian filter (see Supplementary Note 2 for more detail).

Both binning and Gaussian filtering are differentiable in network training, so the ONN weights (phase patterns of the SLMs) could be adaptive to these regularizations during training. Even though the performance in computational results may degrade a little bit because of the regularizations, the difference between simulation results and optical output is decreased. As the pixel sizes of sensors and SLMs were not the same (3.45 μm and 8 μm respectively), we use a homography transformation and linear interpolation (see Supplementary Fig. S12 for calibration details) to warp the sensor images to SLM phase patterns.

### Online training framework for Monet prototype system

In the prototype system, we used two input images of different views and four phase-modulation interference units to generate interference patterns, while eight diffractive units and interference units were iteratively appended for regressing the depth of objects. Due to the non-ideal characteristics of the laser and small misalignments in optical modulation devices, a hybrid optical-electronic online training framework is necessary for Monet to overcome the severe distortion in output depth map. To guarantee that our Monet can work well in physical systems, two adjustments were deployed in this physical system compared with the network we used in Fig. 2: (1) Simplifying the network architecture with fewer feature maps (smaller width) and network layers (smaller depth) to avoid overfitting. (2) Adding one more DU before output to suppress the speckles by regularizing the smoothness of the SLM phase-modulation pattern (see Supplementary Fig. S10 for details). Our Monet prototype system successfully predicted the depth differences of intra-object and among objects physically with proposed online training framework and these network adjustments.

## Supplementary information

Supplementary Information

Monet results on the WHU stereo dataset (3D perception)

Monet results on the VisDrone dataset (moving object detection)

Prototype system and results on the THU dataset

## Data Availability

The data that support the plots within this paper and other finding of this study are available from the corresponding author upon reasonable request.

## Code availability

All relevant code is available from the corresponding author upon reasonable request.
