# Supplementary material for: A multichannel optical computing architecture for advanced machine vision
Source: Light Sci Appl. 2022 Aug 18;11:255. doi: 10.1038/s41377-022-00945-y (PMC9385649; doi:10.1038/s41377-022-00945-y)
Supplement: Supplementary file 1 — Supplementary Information [file 41377_2022_945_MOESM1_ESM.pdf]

Supplementary Information for

A multichannel optical computing architecture for advanced machine vision

Zhihao Xu[1,2,3,†], Xiaoyun Yuan[1,4,†], Tiankuang Zhou[1,3], Lu Fang[1,2,4,*]

[1] Sigma Laboratory, Department of Electronic Engineering, Tsinghua University, Beijing, China.

[2] Beijing National Research Center for Information Science and Technology (BNRist), Beijing, China.

[3] Tsinghua Shenzhen International Graduate School, Shenzhen, China.

[4] Institute for Brain and Cognitive Science, Tsinghua University (THUIBCS), Beijing, China.

[†] These authors contributed equally to this work.

[*] Corresponding author. Email: fanglu@tsinghua.edu.cn.

Outline

Supplementary Fig. S1. Five basic neural network modules of Monet.

Supplementary Fig. S2. Monet architecture for stereo depth estimation.

Supplementary Fig. S3. Network architecture for moving object detection.

Supplementary Fig. S4. Results of stereo depth estimation on WHU stereo dataset.

Supplementary Fig. S5. Results of extra-shift experiment of Monet.

Supplementary Fig. S6. Results of moving object detection on VisDrone dataset.

Supplementary Fig. S7. Quantitative evaluation of moving object detection on VisDrone dataset.

Supplementary Fig. S8. Experimental results of moving object detection on VisDrone dataset.

Supplementary Fig. S9. Quantitative evaluation of moving object detection on VisDrone dataset under physical experiments.

Supplementary Fig. S10. Online training and de-speckling mask.

Supplementary Fig. S11. Results of stereo depth estimation on THU dataset.

Supplementary Fig. S12. SLM-sensor calibration.

Supplementary Fig. S13. Background calibration of the physical system.

Supplementary Fig. S14. SLM modulation calibration.

Supplementary Fig. S15. THU dataset capturing.

Supplementary Fig. S16. Simultaneous depth perception and object recognition.

Supplementary Note 1. Derivation of the phase encoded multi-image interference.

Supplementary Note 2. Optical regularizations for inputs and weights.

Supplementary Note 3. Computing efficiency analysis of Monet.

Supplementary Video 1. 3D visualization of stereo depth estimation.

Supplementary Video 2. Results of moving object detection.

Supplementary Video 3. Physical system for stereo depth estimation.

Supplementary Figures

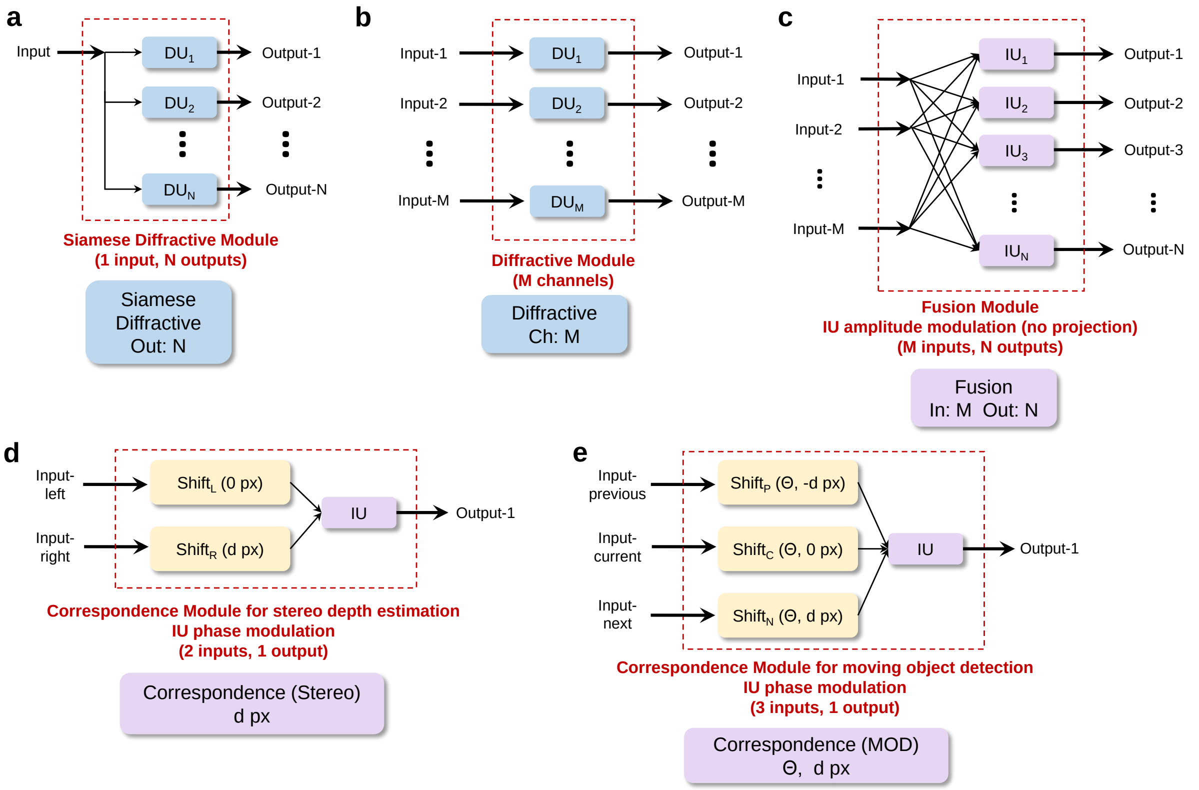

Supplementary Figure S1 | Five basic neural network modules of Monet. The top part of each subfigure shows the detailed network structure of the module, and the bottom part shows the input and output channel numbers and the icon used to represent the module (used in Supplementary Fig. S2 and S3). **a**, Siamese diffractive module. 1 input, N outputs, N diffractive units (DUs) are used to extract feature maps from the single-channel input. **b**, Diffractive module. M input, M outputs, each input channel is processed by a diffractive unit (DU). **c**, Fusion module. M inputs, N outputs, N interference units (IUs, amplitude modulation). Each IU takes M input feature maps and outputs a weighted summed feature map. **d**, Correspondence module for stereo depth estimation. Two inputs (left and right), 1 output. As discussed in the manuscript, in stereo depth estimation, the spatial position of the left channel is fixed, and the right channel is shifted d pixels to right before interference. **e**, Correspondence module for moving object detection (MOD). Three inputs (previous, current, and next), 1 output. As discussed in the manuscript, we fix the spatial position of the current-frame channel, and shift the previous and next frames along with multiple directions. Here, we keep the three channels in a line with the current channel in the center. The projection function can be represented by two parameters, shifting direction Θ and shifting distance d. The next channel is shifted d pixels along the direction Θ, and the previous channel is shifted d pixels along the opposite direction.

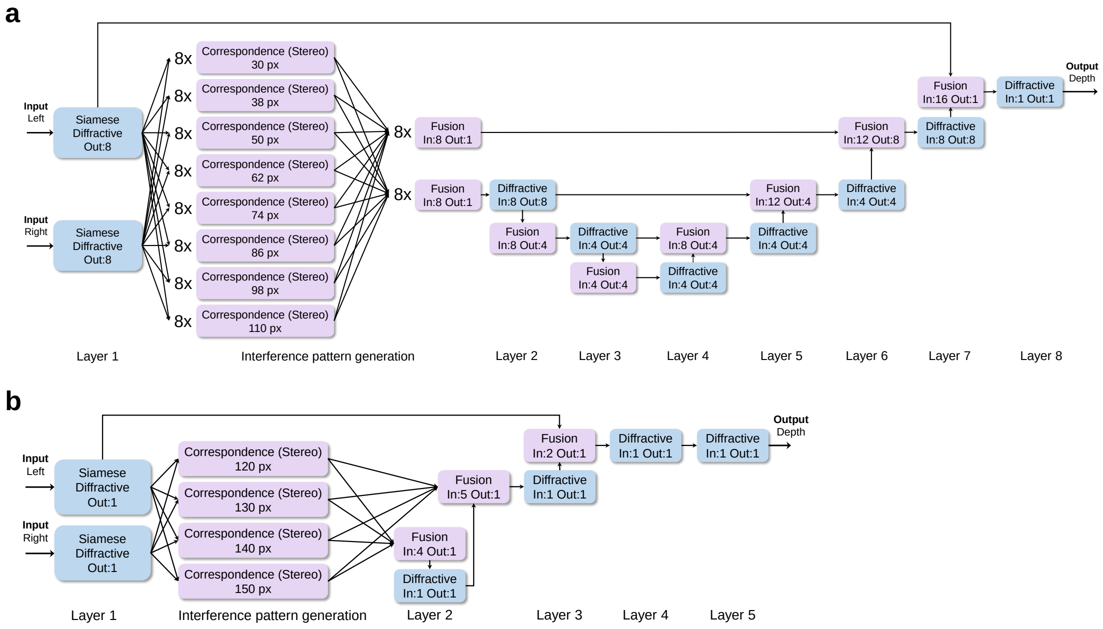

Supplementary Figure S2 | Monet architecture for stereo depth estimation. a, The network architecture for stereo depth estimation on the WHU stereo dataset (Fig. 2). The 8× before the correspondence and fusion module denote that there are 8 same modules to process the 8 channels output by the previous modules. The default (with no number) is 1×. Note that IUs with 8 different shifting distances are adopted to all 8 channels output by Layer 1. Thus, a total of 64 IUs are deployed for interference pattern generation. Sixteen fusion modules (8 input channels, 1 output channel) are followed to process these patterns. **b**, The network architecture for the prototype system on the THU dataset (Fig. 4). Two networks have very similar architectures, but we made two changes: 1) Simplifying the network structure with fewer feature maps and network layers to avoid overfitting. Specifically, 8 shifts with 8 channels are used in the network for the WHU stereo dataset and only 4 shifts with 1 channel are used for the prototype system. The former network also used more channels and layers for feature extraction and depth estimation in the regression module. 2) Adding one more DU (denoted de-speckling mask) before output to suppress the speckles by regularizing the high frequency of the SLM phase-modulation pattern (Supplementary Fig. S7 and S8).

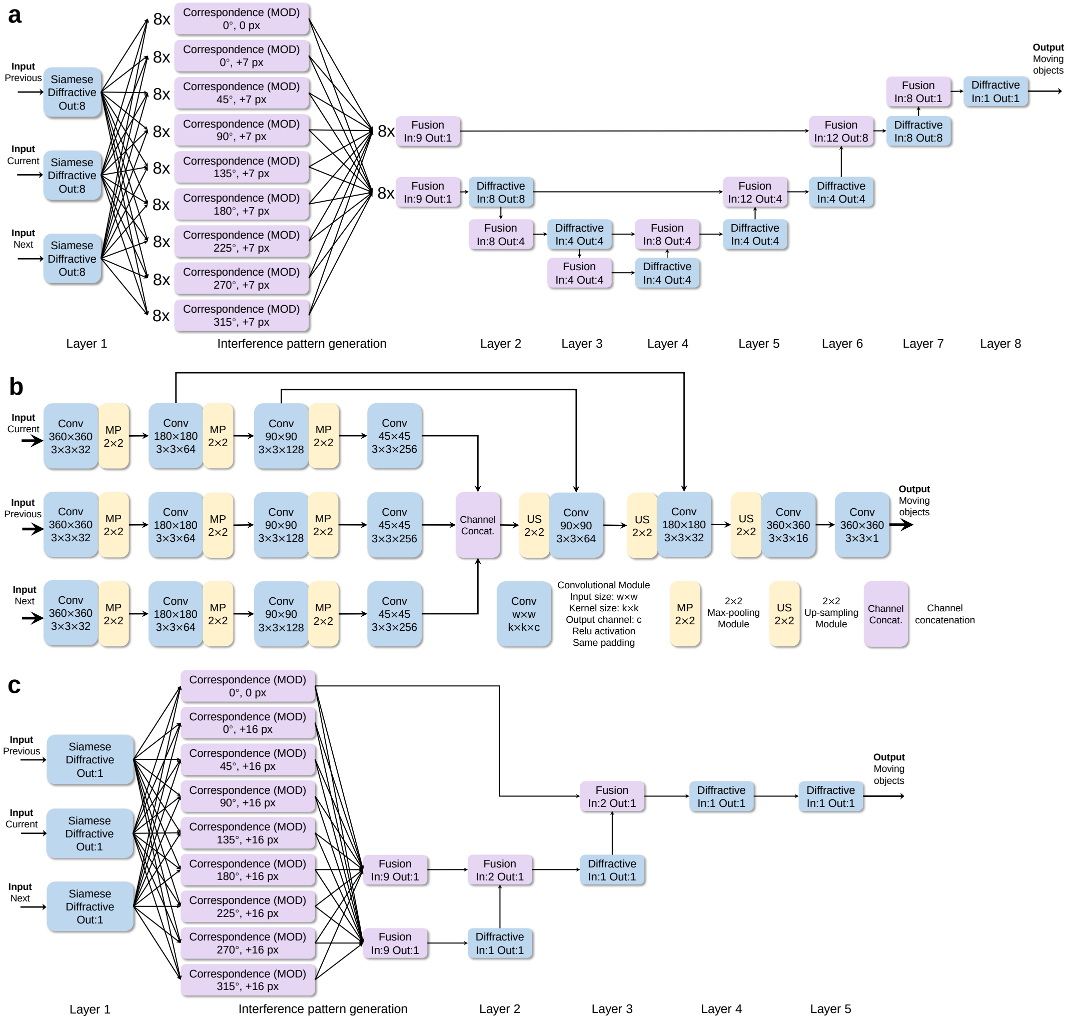

Supplementary Figure S3 | Network architecture for moving object detection. a, The Monet architecture used for moving object detection on the VisDrone dataset. It has three inputs and adopts a 2-direction-shifting function. For the VisDrone dataset, 9 directions with a 7-pixel shifting distance (1 stopped, 8 moving directions from 0° to 315° with a 45° step) are adopted by the projection function. The object regression has the same structure as that used in stereo depth estimation. Similar to Supplementary S2, the 8× before the correspondence and fusion module denote that there are 8 same modules to process the 8 channels output by the previous modules. The default (with no number) is 1×. Note that IUs with 9 different shifting orientations and distances are adopted to all 8 channels output by Layer 1. Thus, a total of 72 IUs are deployed for interference pattern generation. Sixteen fusion modules (9 input channels, 1 output channel) are followed to process these patterns. **b**, Electronic convolution neural network (CNN) used for comparison. The CNN has a similar 3-path network structure as the Monet, and the feature maps from the three inputs are concatenated and input to the regression module for moving object detection. **c,** Monet architecture used for moving object detection with physical experiments. The backbone of the network is same as fig. S3(a). We kept the shifting interference part unchanged except for the shifting pixel changing from 7 to 16 pixels. The reason is that we decided to use all the pixels in SLM, so we combine several SLM pixels to one. The shifting numbers in the figure indicates the actual pixel numbers in SLM. For the output regression part, we reduced the layer amount from 8 to 5 to eliminate overfittings in a small dataset in physical experiments.

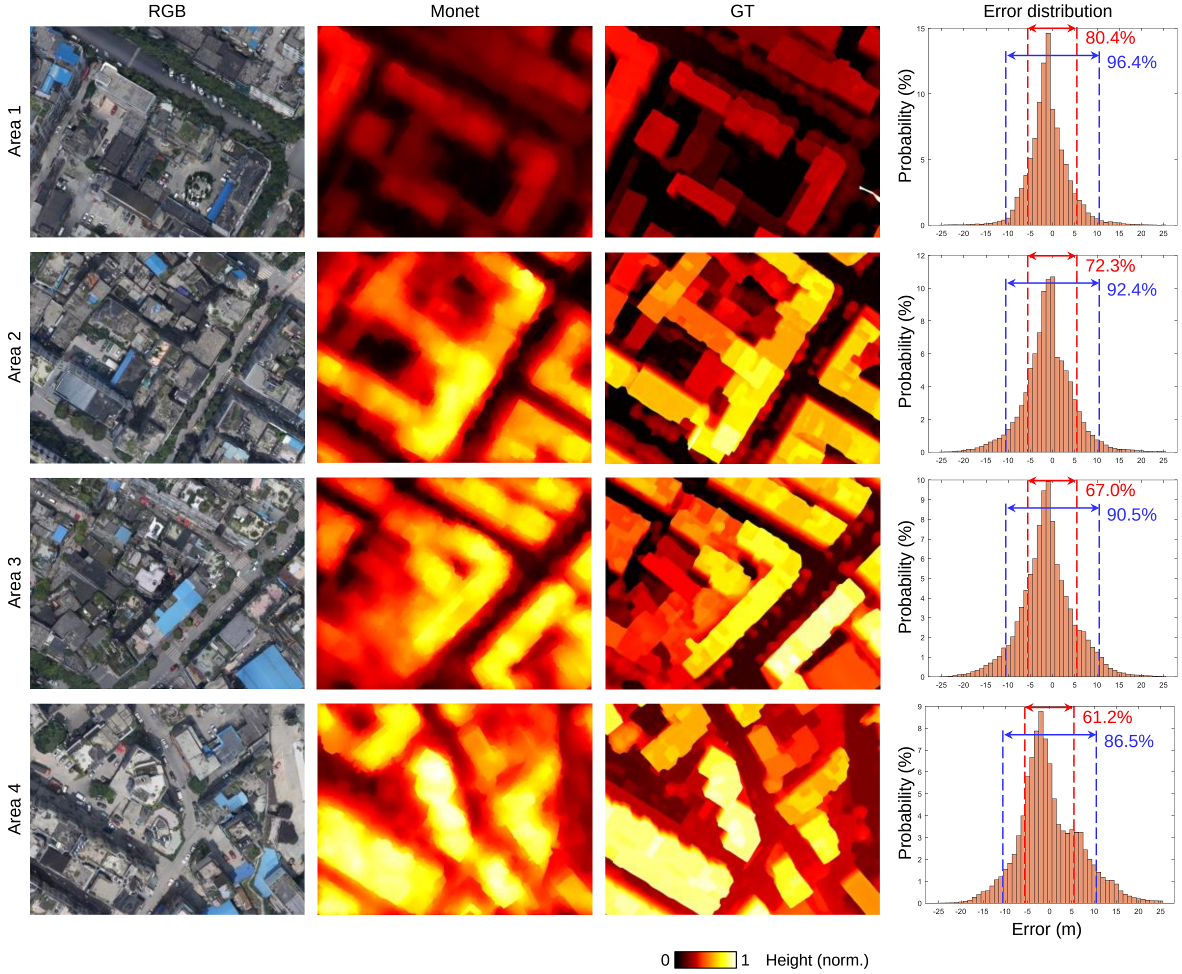

Supplementary Figure S4 | Results of stereo depth estimation on WHU stereo dataset. Depth estimation results of 4 areas in the test set. From left to right, RGB image of the left-view camera, estimated depth map of Monet (converted to heights relative to the ground), the ground-truth depth map, and the error distribution. GT, ground-truth. Norm., normalized.

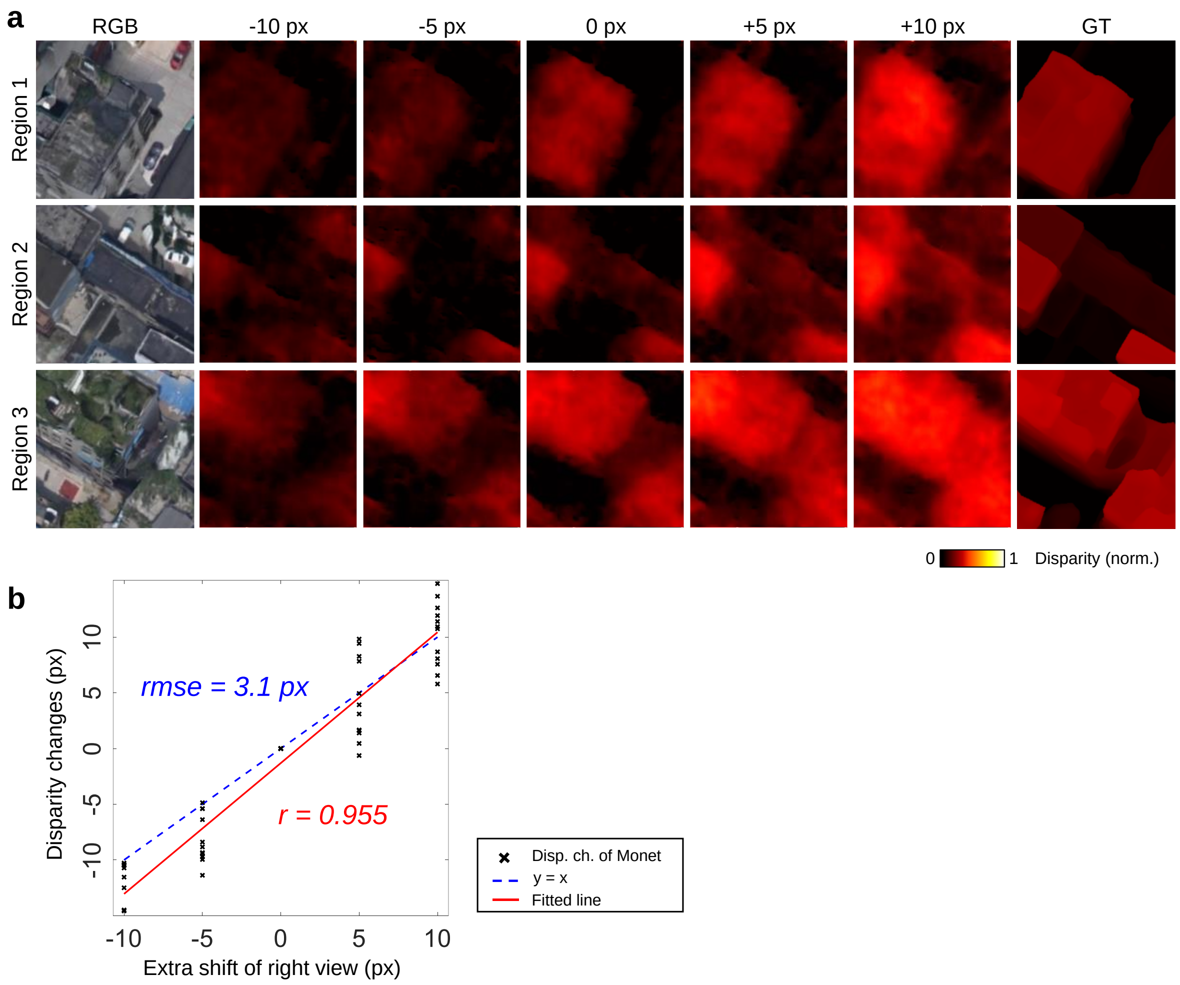

Supplementary Figure S5 | Results of the extra-shift experiment on the WHU stereo dataset. The purpose is to show that Monet predicts the depth information from the disparity of the stereo images, not just learning a style transferring or monocular depth estimation model. In particular, we fix the spatial position of the left-view images (test set only), and apply a series of extra-shifts (-10, -5, 0, 5, 10 pixels to left) on the corresponding right-view images to form a new test set. If Monet learns how to estimate the depth information, the pixel value of the output disparity map will change along with the extra shifts. **a**, Output depth information (rendered as disparity value) of three representative regions from the new test set. The disparity maps of the same region are normalized using the same scale and rendered using the same colormap. The Monet results show that with the increasing of the extra shift, the pixel values on the disparity map also become larger. **b**, Quantitative analysis results. We randomly sample 15 small regions (20×20 pixels) from these 3 regions, and plot the disparity values relative to the baseline using black crosses (the disparity value with 0 extra shift is used as the baseline). These relative disparity data points are further fitted using a red line, showing a strong correlation (r=0.955). The blue dash line represents the ground-truth changes of the relative disparity. The RMSE between the output of Monet and ground-truth is 3.1 px. GT, ground-truth. Norm., normalized. Disp., disparity. Ch., changes. Px, pixel.

48

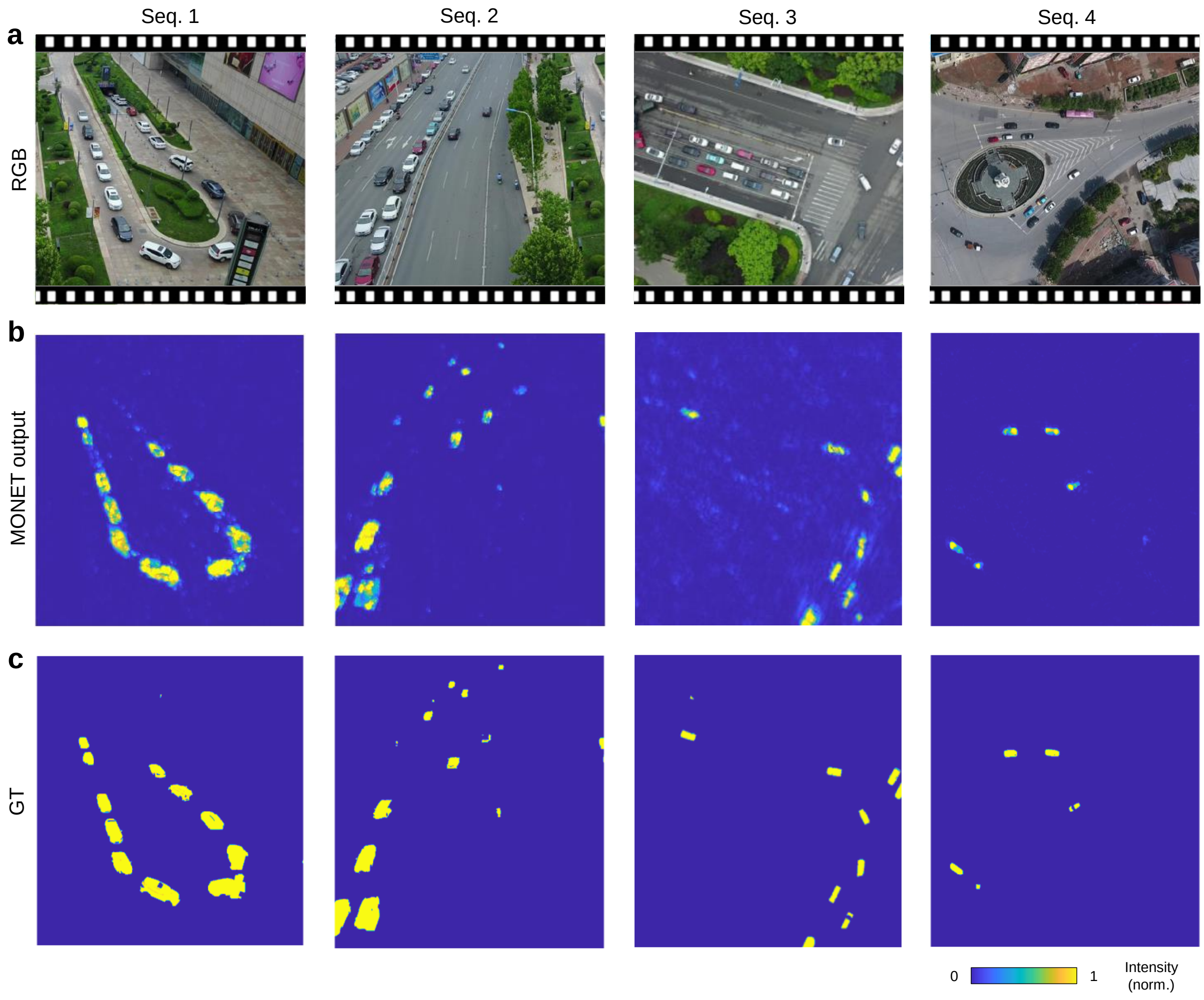

Supplementary Figure S6 | Results of moving object detection on VisDrone dataset. a, Representative frames of the 4 video sequences in the test set. **b**, Monet computational output of the four video sequences (sequence 1 & 4 had already been presented in the manuscript). **c**, Ground truth moving object mask of each video sequence. We used one set of weights to predict all the output of these 4 sequences. The scales and backgrounds of each scene were varying but our Monet could successfully predict the moving object mask, which indicated the generalization ability of our Monet structure. GT., ground truth. Seq., sequence.

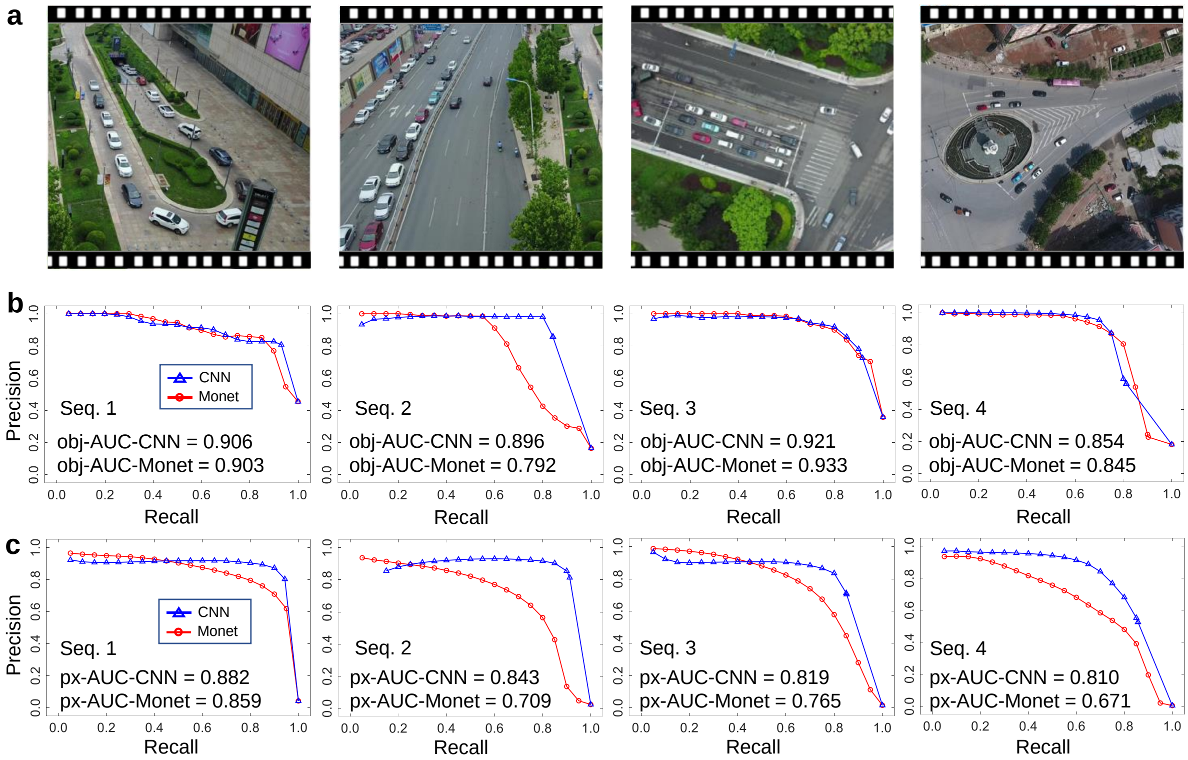

Supplementary Figure S7 | Quantitative evaluation of moving object detection on VisDrone dataset. a, Representative frames of the 4 video sequences in the test set. **b**, Object-level PR curves and the AUCs of the 4 video sequences (already presented in the manuscript). **c**, Pixel-level PR curves and the AUCs of the 4 video sequences. Both Monet and CNN performed worse in terms of pixel-level than object-level, but the degradation of CNN is smaller. The reason is that our Monet did not generate very sharp edges in the outputs, leading to low pixel-level performance. Seq., sequence.

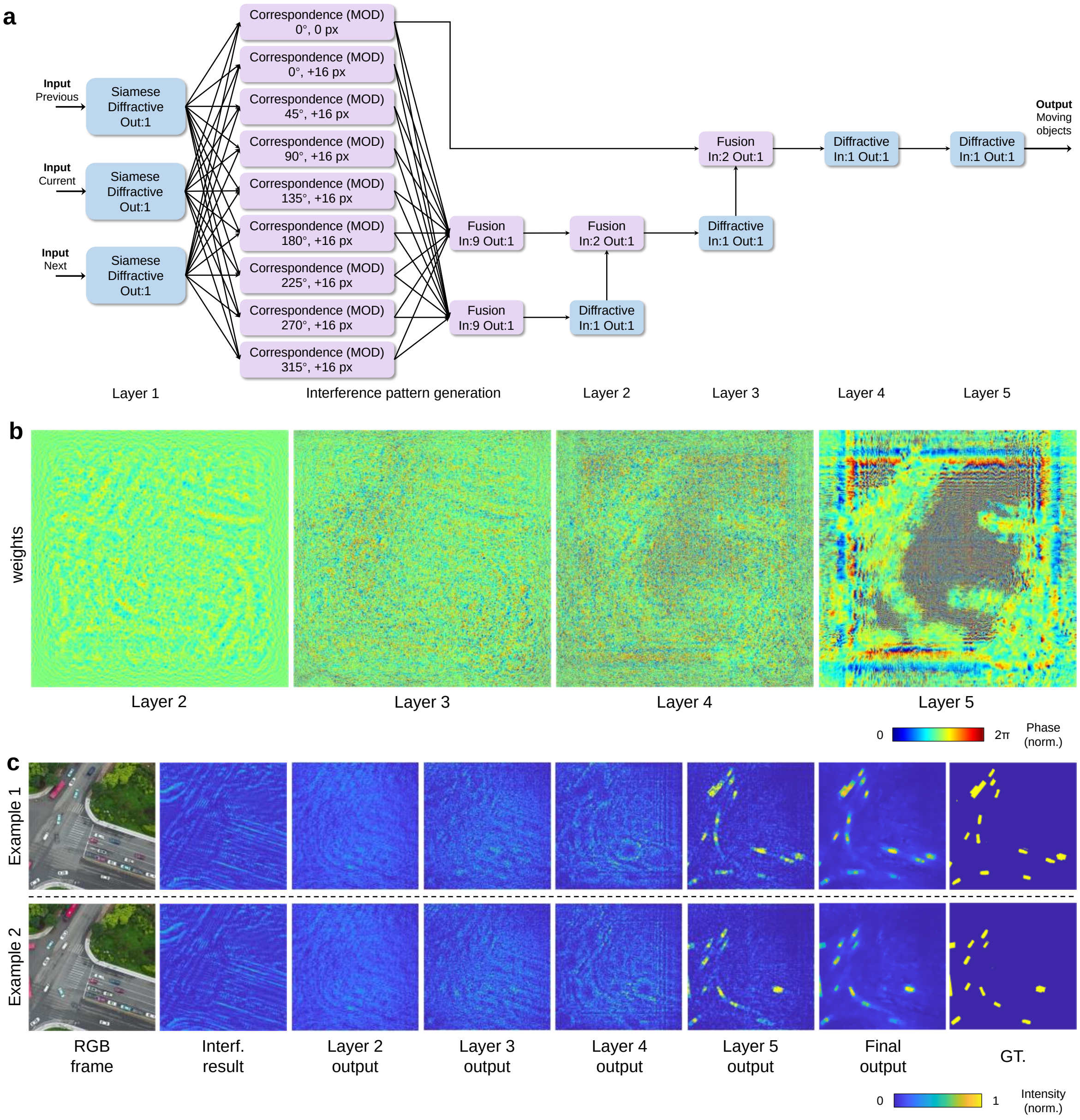

Supplementary Figure S8 | Experimental results of moving object detection on VisDrone dataset. a, The network structure of Monet in the physical setup. **b,** Trained phase map (weight) of the DU in layer 2, 3, 4, and 5. Note that we regard the shifting interference as layer 1, so the first DU occurs in layer 2. **c,** The intermediate results of two representative frames in the test video. Similarly, to get a better visualization result, we applied the bilateral solver on the final output to get rid of the speckles and noise caused by the laser property and calibration error of the optical components.

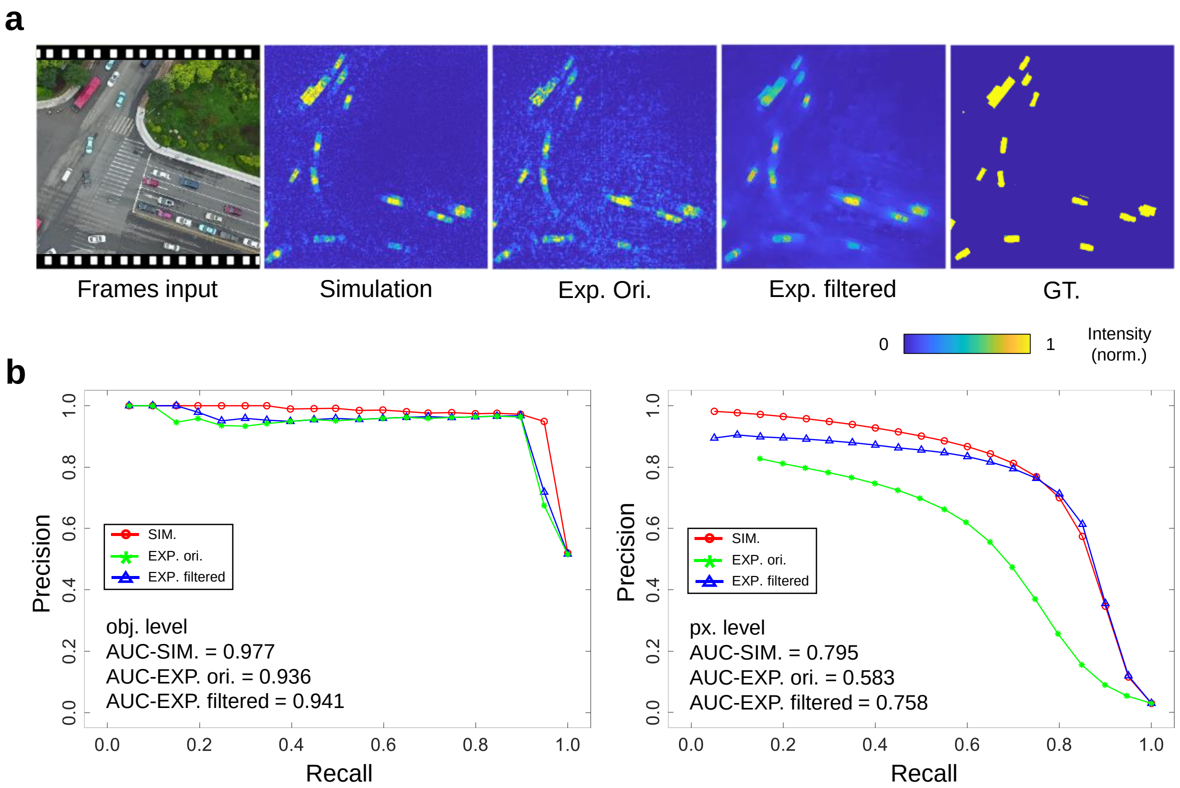

Supplementary Figure S9 | Quantitative evaluation of moving object detection on VisDrone dataset under physical experiments. a, Representative frame, simulation result, original optical output, filtered optical output and ground truth moving object mask of the test video sequence. **b**, Object-level PR curves and the AUCs of the test video sequence (already presented in the manuscript) and pixel-level PR curves and the AUCs of it. The optical output showed comparative results with simulation results in object level. However, due to the speckles of laser and noise, the pixel level metrics are degraded compared with simulations. To get better visualization, we could apply a bilateral filter for the final optical output for denoising. This action did not affect the object level performance but increased the pixel level performance due to the elimination of noise. Note that the simulations and filtered optical output showed comparative results in pixel-level benchmarks. Sim., simulation. Exp., experimental. Ori., original. Obj., object. Px., pixel. Norm., normalization.

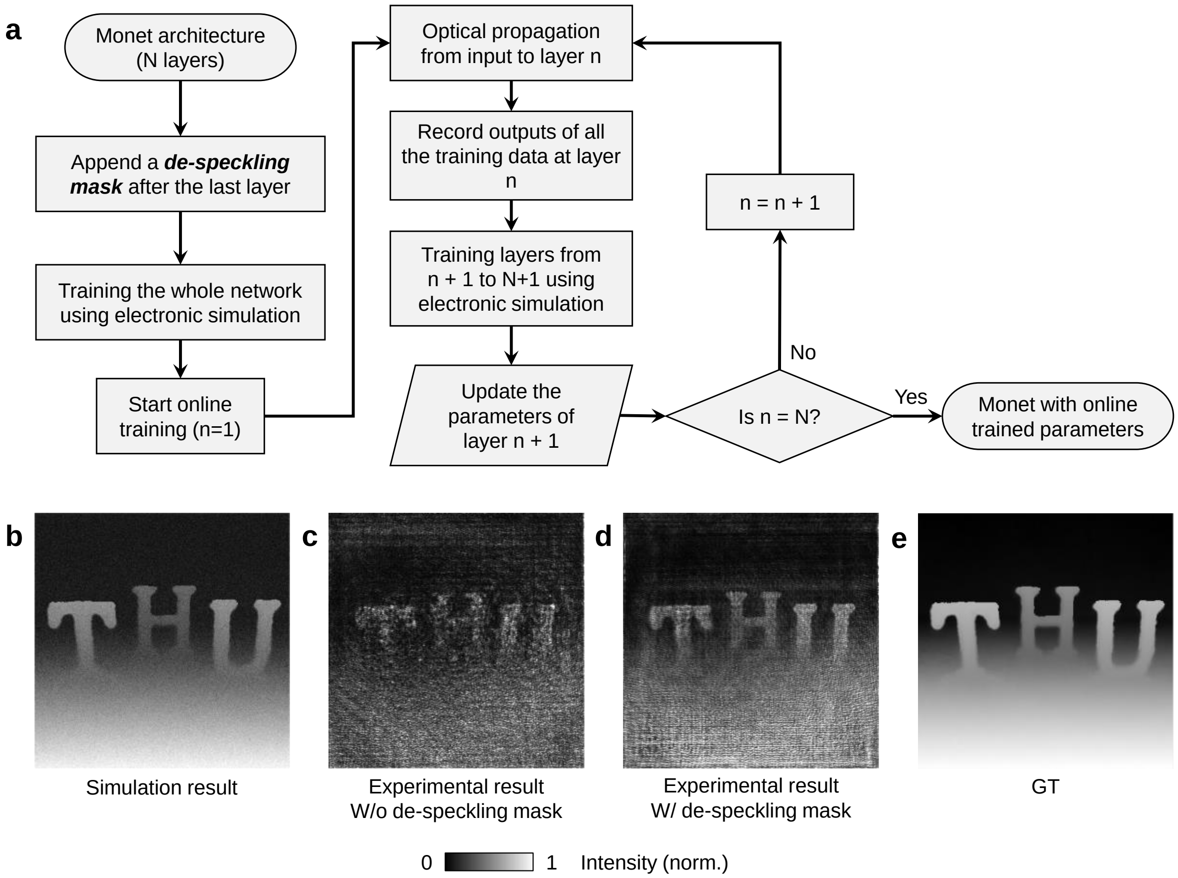

Supplementary Figure S10 | Online training framework and de-speckling mask of Monet prototype system. a, Online training framework. Due to the non-ideal laser and optical modulation devices, directly copying the network parameters trained using electronic simulation to the prototype system will dramatically degrade the performance, resulting in a severely distorted depth map with nearly no useful information. The proposed online training framework overcomes this problem by adopting hybrid optical and electronic training. Specifically, online training starts from the first layer, records the outputs of all the training data of the first layer, and the outputs are used as new training data to finetune the parameters from the second layer to the last layer. Then, we record the outputs of the second layers using the finetuned parameters, and repeat the process until the last layer. Besides, an extra de-speckling mask layer is appended at the end of the neural network. The de-speckling mask layer is implemented using a single DU with smoothed phase mask. In the electronic simulation, a Gaussian filter (σ=11) is applied to the phase mask before it is used to modulate the wavefront. A smooth phase mask can effectively reduce the artifacts (Subfig. c) caused by the small misalignment of the optical modulation devices, leading to a more visually pleasant result (Subfig. d). **b**, Simulation result. The simulation result is almost perfect with few speckle artifacts as the laser wavefront and the optical modulation devices are ideal (purely flat) in simulation. **c**, Optical results w/ online training but w/o de-speckling mask. With online training, the three character-shaped objects ‘T’, ‘H’, ‘U’ can be figured out in the output depth map. However, there are very severe speckle artifacts caused by the small misalignment of the optical modulation devices. **d**, Optical results w/ online training and w/ de-speckling mask. The speckle artifacts are effectively suppressed. **e**, Ground-truth depth map. W/o, without. W/, with. GT, ground-truth.

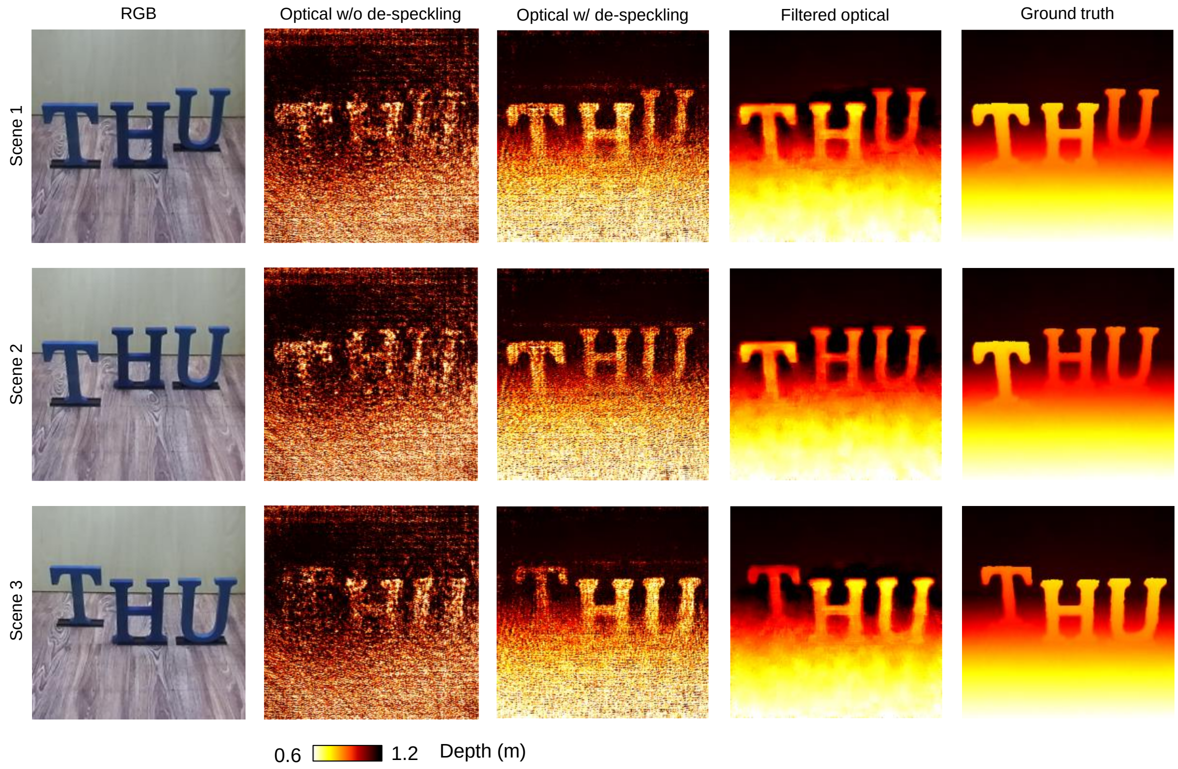

Supplementary Figure S11 | Results of stereo depth estimation on THU dataset. Depth estimation results of 3 scenes in the THU test set. From left to right, RGB images of the left-view camera, optical results (experimental results of the prototype system) w/o de-speckling mask, optical results w/ de-speckling mask, filtered optical results using bilateral solver and the ground-truth depth maps. W/o, without. W/, with. GT, ground-truth.

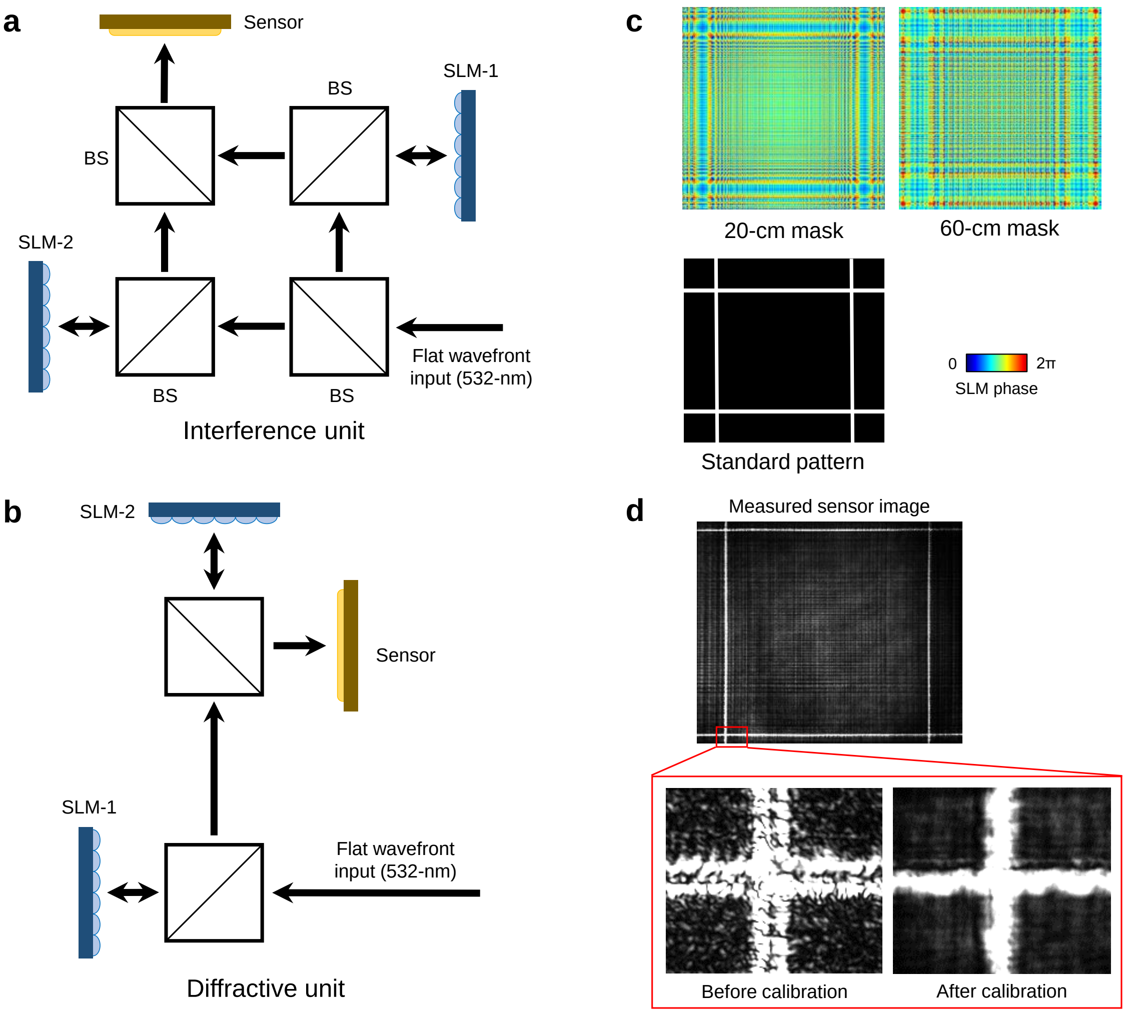

Supplementary Figure S12 | SLM-sensor calibration. The purpose of calibration is to align the two SLMs and the sensors. **a**, Optical configuration of the two-input interference unit. The distance from both SLMs to the sensor is 20 cm. **b**, The optical configuration of the diffractive unit. SLM-1 is used to encode the input image, and SLM-2 is used for phase modulation. The distance from SLM-1 to the sensor is 60 cm and the distance from SLM-2 to the sensor is 20 cm. **c**, Two trained phase masks for system calibration. The masks will modulate the flat wavefront and generate a standard pattern at the designed distance (20 cm and 60 cm). The pattern is shown in the bottom left: a square formed by two horizontal and two vertical lines. In prototype system implementation, we first adjust the position and pose of the SLM and sensor to generate a sharp/focused pattern (a sharp square) on the sensor plane. This will ensure that the SLM plane is parallel to the sensor plane and the physical distance is close to the designed distance. The next step is to adjust the two SLMs in x-y plane to make the two patterns generated by the SLMs fully overlapped. This will ensure that the two SLMs are aligned in the x-y plane. **d**, Patterns captured by the sensor. After calibration, the two patterns are fully overlapped. The resolution of the CMOS sensor is 2448×2048 with a 3.5-μm pixel size, while our simulation resolution is 800×800 with an 8-μm pixel size. We detected the 4 corners of the captured pattern and the standard pattern, and estimate a homography transformation matrix from the sensor image to the standard pattern (bottom left of subfig. c). After homography transformation, the resolution and pixel size of the sensor image will match the simulation setting. BS, beam splitter, SLM, spatial light modulator.

84

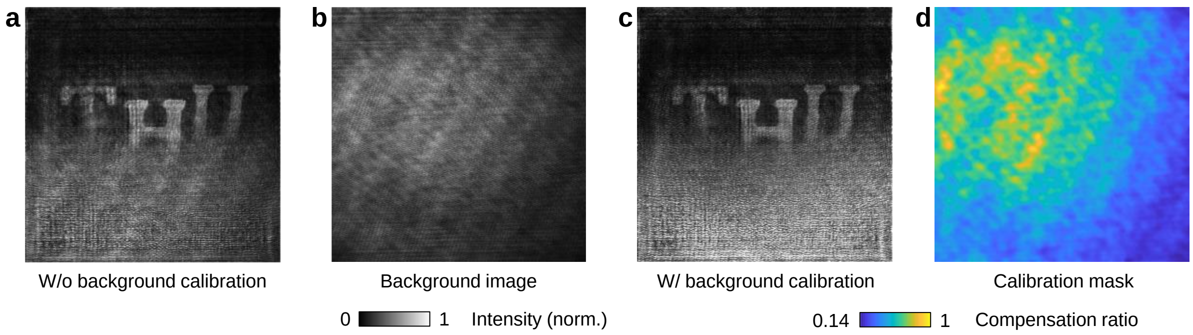

Supplementary Figure S13 | Background calibration of the physical system. Due to the non-ideal optical modulation devices and laser, there are artifacts in the results, and the background is non-uniform. The purpose of background calibration is to remove them. **a**, Raw result captured by the sensor. There are obvious periodical stripes in the bottom part of the image. **b**, The captured background image with no phase modulation on both SLMs. Similar stripes also appear. The background image is then smoothed (Gaussian smooth, σ=5, mask size, 800×800), normalized by dividing its maximum value, and used as the calibration mask (Subfig. d). **c**, Image with background calibration. We divided the raw image values by the calibration mask (Subfig. d) pixel-wisely to get the artifact-free image. The stripes were successfully removed. **d**, The calibration mask. W/o, without. W/, with. Norm., normalized.

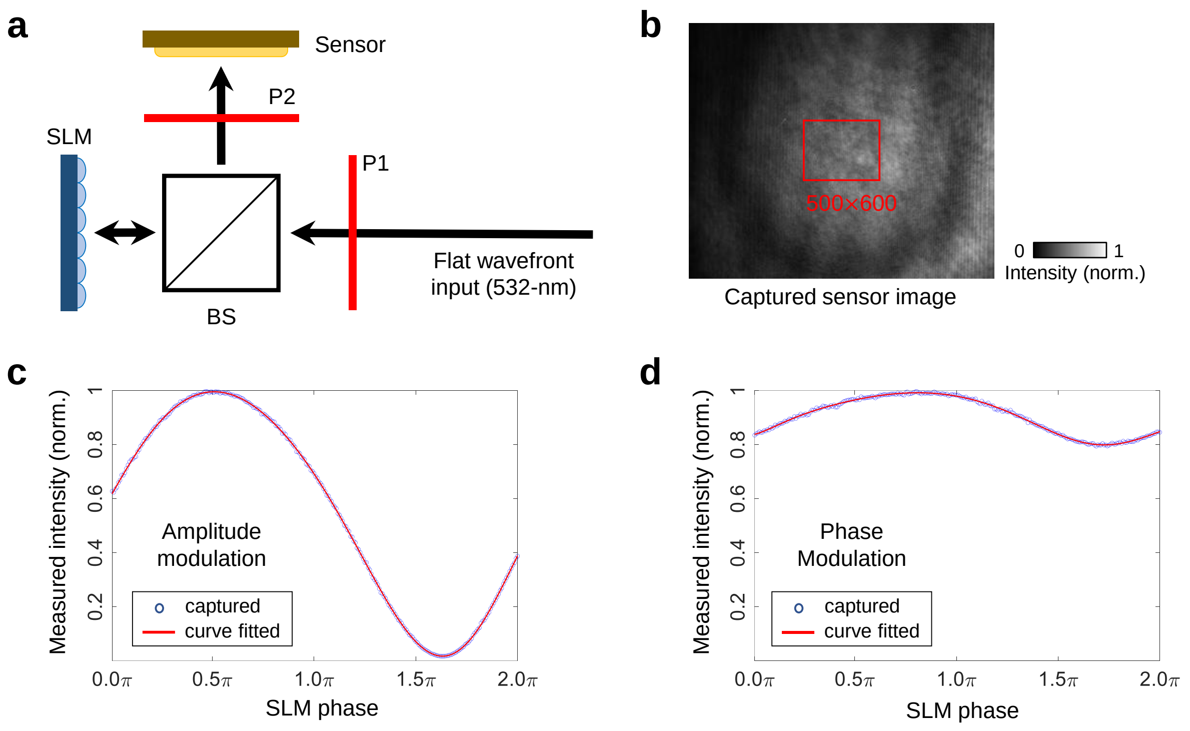

Supplementary Figure S14 | SLM modulation calibration. a, The optical configuration of SLM modulation calibration. A flat 532-nm wavefront is used as the input. Two polarizers P1 and P2 are placed before and after the SLM. As the SLM will change both the phase and the polarization direction of the wavefront, P2 will change the amplitude of the modulated wavefront. By adjusting the polarization direction of P1 and P2, SLM can achieve amplitude modulation or phase modulation. In amplitude modulation, P1 is adjusted +45° to the fast axis of SLM, and P2 is adjusted -45° to the fast axis. In phase modulation, both P1 and P2 are adjusted parallel to the fast axis of SLM. The relation between the input phase and the output amplitude (measured using the optical field intensity) is calibrated using a sensor and fitted using 10th order polynomial functions. **b**, The captured sensor image. We scan the SLM modulation phase from 0 to 2π (256 steps), and record the sensor image. The average intensity of the center 500×600 region is used for calibration. **c**, Phase-intensity relation of the amplitude modulation mode. **d**, Phase-intensity relation of the phase modulation mode. Two 10th order polynomial functions are used to fit the two curves. In the amplitude modulation mode, the intensity changes obviously with the modulation phase. The ratio of maximum intensity and minimum intensity is 59.98 (17.78 dB). While in the phase modulation mode, the intensity change is much smaller (1.26, ≈1 dB). BS, beam splitter. SLM, spatial light modulator. P, polarizer. Norm., normalized.

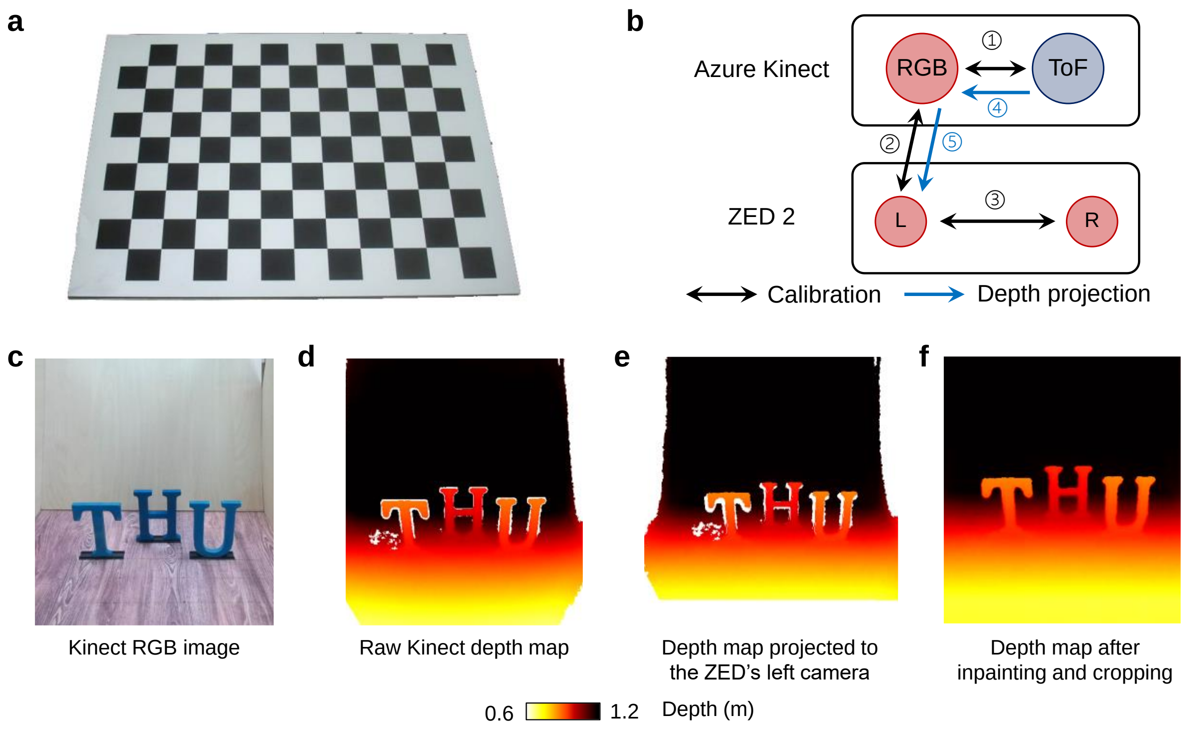

Supplementary Figure S15 | THU dataset capturing. a, Chessboard for calibrating the hybrid camera system. **b**, Calibration and depth mapping pipeline. The RGB and ToF cameras inside the Azure Kinect are pre-calibrated in the factory. The RGB camera inside the Azure Kinect and the left-view camera of ZED 2 are calibrated using the chessboard. The left-view and right-view cameras of the ZED 2 are also calibrated using the chessboard. With the calibration parameters (intrinsic and extrinsic camera matrices), the depth map captured by the ToF camera inside the Azure Kinect can be projected to the RGB camera inside the Azure Kinect using the function provided by the Microsoft Azure SDK. The depth map can be further projected to the left-view camera of the ZED 2 as the ground-truth label using the calibrated camera matrices. **c**, RGB image captured by the left-view camera of the ZED 2. **d**, Raw depth map captured by the ToF camera inside the Azure Kinect. The holes are caused by the imperfect reflection of the light. **e**, Depth map projected to the left-view camera of the ZED 2. The holes become larger due to the viewpoint difference between the ToF camera and the left-view camera of the ZED 2. **f**, Depth map after inpainting and cropping. The holes are well filled.

89

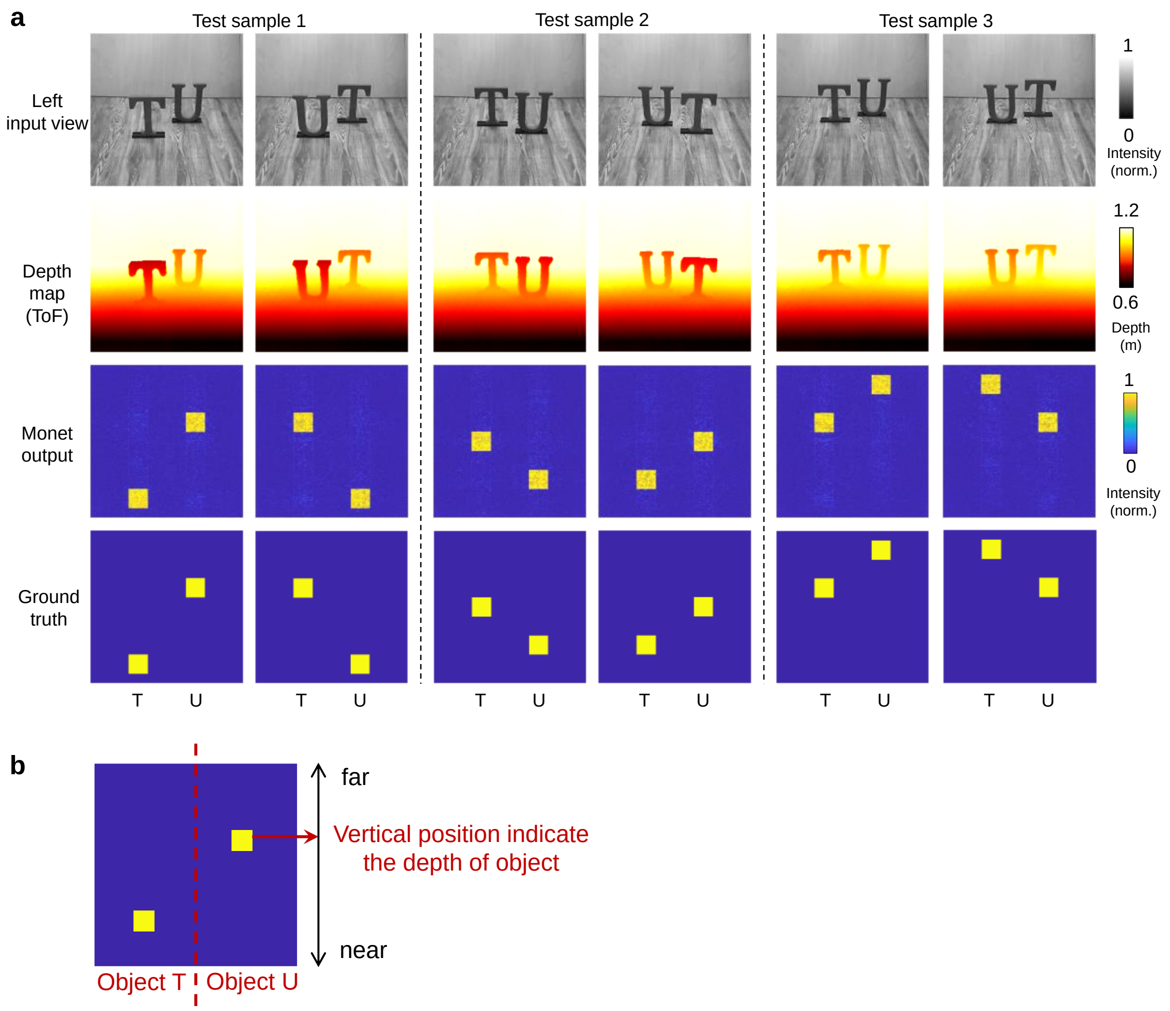

Supplementary Figure S16 | Simultaneous depth perception and object recognition. To further demonstrate the 3D detection as well as intelligent computing ability of Monet, we designed a simultaneous depth perception and object recognition task. In this experiment, we moved each object in 7 positions from near to far. In this way, there are 7x7x2 = 98 samples available for training and testing. We randomly selected 4 samples and their position-exchanged samples (8 in total) among them as the testing data. Here 3 test samples are illustrated. Our Monet successfully recognizes the objects (intelligent computing) and estimates their depth (sensing) simultaneously. Moreover, in Monet, we could achieve abundant intelligent computing tasks by designing the labels for training. However, LiDAR or RGBD cameras only have the depth estimation ability. **a,** First row, Left input view. Second row, depth map captured by Azure Kinect (ToF based). Lidar is also based on the ToF technique, so its outputs will be very close to the second raw, but much sparser. ToF-based cameras/sensors can get very accurate depth value of the pixels in the FoV, but it can not detect and recognize the objects in the scene. Third row, output of our Monet. Fourth row, ground truth labels. **b,** The designed labels for Monet training. The vertical positions of the square with highest intensity indicates the depth value of the object. Regardless of the relative position of the object T and object U, the left square in the output image always represents the depth of the object T and the right square always represents the depth of the object U. Norm., normalized.

Supplementary Notes

Supplementary Note 1 Derivation of the phase encoded multi-channel interference

$$e^{j\mathbf{Y}_1} + e^{j\mathbf{Y}_2} + \cdots + e^{j\mathbf{Y}_N} = (\cos \mathbf{Y}_1 + \cos \mathbf{Y}_2 + \cdots \cos \mathbf{Y}_N) + j(\sin \mathbf{Y}_1 + \sin \mathbf{Y}_2 + \cdots \sin \mathbf{Y}_N)$$

$$\begin{aligned} \left|e^{jY_1} + e^{jY_2} + \cdots + e^{jY_N}\right| &= (\cos \mathbf{Y}_1 + \cos \mathbf{Y}_2 + \cdots \cos \mathbf{Y}_N)^2 + (\sin \mathbf{Y}_1 + \sin \mathbf{Y}_2 + \cdots \sin \mathbf{Y}_N)^2 \\ &= \sum_{i=1}^{N} (\cos^2 \mathbf{Y}_i + \sin^2 \mathbf{Y}_i) + 2\sum_{i}^{N}\sum_{j+1}^{N} \left(\cos \mathbf{Y}_i \cos \mathbf{Y}_j + \sin \mathbf{Y}_i \sin \mathbf{Y}_j\right) \\ &= N + 2\sum_{i}^{N}\sum_{j+1}^{N} \cos\left(\mathbf{Y}_i - \mathbf{Y}_j\right) \end{aligned}$$

Supplementary Note 2 Optical regularizations for inputs and weights

For the modulation SLM (SLM-2 in Fig. 1), we apply a Gaussian filter on the phase map. For the sensor, we use the pixel binning function. This binning action serves as the optical averaged pooling operation to replace the outstanding values with the averaged neighbor pixel values which is commonly adopted in conventional electronic neural networks. In our experiments, binning size is set to 8. For the patterns which encode the network weights, we set the sigma value equal to 9 or 11 and the filter size equal to 4 times of the sigma.

Supplementary Note 3 Computing efficiency analysis of Monet

In terms of computing efficiency, Monet is composed of IUs and DUs, where each N-channel IU consists of N diffractive propagations, N complex field summations, and 1 intensity activation, while each DU consists of 2 diffractive propagations, 1 phase modulation, and 1 intensity activation. As noted in [1], for spatial resolution R by R, each diffractive propagation takes $R^2 \times (4 \times R^2 - 1) = 4 \times R^4 - R^2$ real operations, each complex filed summation takes $2 \times R^2$ real operations, and both phase modulation and intensity activation take $6 \times R^2$ real operations. Hence each N-channel IU takes $N \times 4 \times R^4 + N \times R^2 + 6 \times R^2$ real operations and each DU takes $8 \times R^4 + 10\, R^2$ real operations. In our implementation, the 8-layer Monet (see Supplementary Fig. S3a) is composed of 72 3-channel IUs, 4 4-channel IUs, 9 8-channel IUs, 16 9-channel IUs, 12 12-channel IUs, and 57 DUs. It indicates that there need $2368 \times R^4 + 1270 \times R^2$ real operations for IUs and $456 \times R^4 + 570 \times R^2$ real operations for DUs. Given

that the spatial resolution is 400×400 (R = 400), Monet relates to **72.29 TOPs** (real operations) in total.

The inference speed of Monet is mainly bounded by the frame rates of SLMs and sensors. The updating speed of SLM could reach 1436 FPS [2]. A fast sensor such as Andor Zyla sCMOS sensor supporting ~145 FPS with a 400×400-pixel region [3]. Hence, the inference speed of each IU or DU could reach 0.0068s per frame (145 FPS). Considering the parallel computing ability of Monet, all the operations in one layer could be done simultaneously. Each Monet layer consists of 1 IU layer followed by 1 DU layer, taking 0.0068×2 = 0.0136s for inference. For an 8-layer Monet, the total inference time would be 0.0136×8 = 0.1088s, leading to a computing power of 72.29 TOPs / 0.1088s = **670.22 TOPs/s**.

In terms of power consumption, only laser sources, SLMs and sensors need power supplies and their powers are 10 Watts, 20 Watts, and 25 Watts, respectively. We also need a computer to control these SLMs and sensors, taking no more than 200 Watts. Each N-channel IU needs 1 laser source, N SLMs, and 1 sensor, and thus takes $35 + 20 \times N$ Watts. Each DU needs 1 laser source, 2 SLMs, and 1 sensor, and thus takes 75 Watts. As the Monet structure mentioned above, all IUs (72 3-channel IUs, 4 4-channel IUs, 9 8-channel IUs, 16 9-channel IUs, and 12 12-channel IUs) will take 15795 Watts×0.0068s = 107.41 J and all DUs (57 DUs in total) will take 4275 Watts×0.0068s = 29.07 J, while the controlling computer will keep running and take 200 Watts×0.1088s = 21.76 J. In this way, the total energy consumption will be 158.24 J, leading to an energy efficiency of 72.29 TOPs / 158.24 J = **0.457 TOPs/J**. Comparatively, for an NVIDIA GTX 1080 Ti GPU at maximum power, the computing capacity is **11.34 TOPs/s** (float-point operations) and the power is 250 Watts TDP (**0.045 TOPs/J**).

Benefiting from the highly-paralleled property of IU and DU computing, the instantaneous power may exceed GPU, however, the energy efficiency of Monet surpasses GPU by one order of magnitude in specific neural network inference tasks. At the current stage, Monet is running with off-the-shelf optical components, but if the Monet architecture was integrated into the photonic chip, the power consumption including laser, modulations, and so on would further decrease, achieving a much higher energy efficiency computing.

[1] Zhou, T., Lin, X., Wu, J., Chen, Y., Xie, H., Li, Y., ... & Dai, Q. (2021). Large-scale neuromorphic optoelectronic computing with a reconfigurable diffractive processing unit. *Nature Photonics*, *15*(5), 367-373.

[2] https://www.meadowlark.com/1024-x-1024-slm/

[3] https://andor.oxinst.com/products/scmos-camera-series/zyla-4-2-scmos
